# Prolyl Oligopeptidase from the Blood Fluke *Schistosoma mansoni*: From Functional Analysis to Anti-schistosomal Inhibitors

**DOI:** 10.1371/journal.pntd.0003827

**Published:** 2015-06-03

**Authors:** Pavla Fajtová, Saša Štefanić, Martin Hradilek, Jan Dvořák, Jiří Vondrášek, Adéla Jílková, Lenka Ulrychová, James H. McKerrow, Conor R. Caffrey, Michael Mareš, Martin Horn

**Affiliations:** 1 Institute of Organic Chemistry and Biochemistry, Academy of Sciences of the Czech Republic, Prague, Czech Republic; 2 First Faculty of Medicine, Charles University, Prague, Czech Republic; 3 Institute of Parasitology, University of Zurich, Zurich, Switzerland; 4 Institute of Molecular Genetics, Academy of Sciences of the Czech Republic, Prague, Czech Republic; 5 Institute of Parasitology, Biology Centre, Academy of Sciences of the Czech Republic, Ceske Budejovice, Czech Republic; 6 Faculty of Science, Charles University, Prague, Czech Republic; 7 Center for Innovation and Discovery in Parasitic Diseases, Department of Pathology, University of California, San Francisco, San Francisco, California, United States of America; René Rachou Research Center, BRAZIL

## Abstract

**Background:**

Blood flukes of the genus *Schistosoma* cause schistosomiasis, a parasitic disease that infects over 240 million people worldwide, and for which there is a need to identify new targets for chemotherapeutic interventions. Our research is focused on *Schistosoma mansoni* prolyl oligopeptidase (SmPOP) from the serine peptidase family S9, which has not been investigated in detail in trematodes.

**Methodology/Principal Findings:**

We demonstrate that SmPOP is expressed in adult worms and schistosomula in an enzymatically active form. By immunofluorescence microscopy, SmPOP is localized in the tegument and parenchyma of both developmental stages. Recombinant SmPOP was produced in *Escherichia coli* and its active site specificity investigated using synthetic substrate and inhibitor libraries, and by homology modeling. SmPOP is a true oligopeptidase that hydrolyzes peptide (but not protein) substrates with a strict specificity for Pro at P1. The inhibition profile is analogous to those for mammalian POPs. Both the recombinant enzyme and live worms cleave host vasoregulatory, proline-containing hormones such as angiotensin I and bradykinin. Finally, we designed nanomolar inhibitors of SmPOP that induce deleterious phenotypes in cultured schistosomes.

**Conclusions/Significance:**

We provide the first localization and functional analysis of SmPOP together with chemical tools for measuring its activity. We briefly discuss the notion that SmPOP, operating at the host-parasite interface to cleave host bioactive peptides, may contribute to the survival of the parasite. If substantiated, SmPOP could be a new target for the development of anti-schistosomal drugs.

## Author Summary

Schistosomiasis (bilharzia) is a major global health problem caused by the schistosome flatworm which lives in the bloodstream. Treatment and control of the disease relies on a single drug, and should resistance emerge, there would be increased pressure to discover new drug targets. Proteolytic enzymes are fundamental to the survival of parasites, and, hence, are attractive targets for drug intervention. Oligopeptidases from the S9 family are known virulence factors for protozoan trypanosomatids but have yet to be studied in parasitic flukes. We, therefore, investigated prolyl oligopeptidase in *Schistosoma mansoni* (SmPOP) and found that it is expressed in those developmental stages that infect humans. We provide a comprehensive analysis of the peptidase’s expression, localization and functional biochemical properties. Interestingly, SmPOP, which is found in the tegument and parenchyma of the parasite, can cleave blood peptides involved in vasoregulation and we discuss how this ability may aid the parasite’s survival. Finally, we designed potent inhibitors of SmPOP that cause deleterious changes in cultured parasites. We conclude that SmPOP is important for parasite survival and may be a potential target for the development of anti-schistosomal drugs.

## Introduction

Schistosomiasis, also known as bilharzia, is caused by blood flukes of the genus *Schistosoma* with approximately 240 million people infected [1]. Three species of schistosome principally infect humans: *Schistosoma haematobium*, which causes urinary schistosomiasis, and *S*. *japonicum* and *S*. *mansoni*, which cause intestinal schistosomiasis [2]. Adult schistosomes can reside for decades as pairs in the veins surrounding the bladder or in mesenteric and the portal veins, and produce hundreds of eggs per day [3]. Morbidity arises from immuno-pathological reactions to and entrapment of schistosome eggs in various tissues [4]. Disease symptoms include spleno- and hepatomegaly, periportal fibrosis and hypertension, and urinary obstruction. Bladder carcinoma, sterility, malnutrition, and developmental retardation are common [3]. Infections can last a lifetime [5].

In the absence of a vaccine [6], control and treatment of schistosomiasis rely on a single drug, praziquantel, and the possibility of emergent drug resistance is a constant concern [7,8]. Accordingly, there is a continued impetus to identify new schistosome drug targets and chemotherapeutically active anti-schistosomals [8,9].

Proteolytic enzymes (peptidases) of schistosomes are attractive drug targets as they operate at the host–parasite interface, where they may facilitate parasite invasion, migration, nutrition and immune evasion [10–12]. Most studies concerning schistosome peptidases have focused on either the serine peptidase called cercarial elastase that facilitates penetration of the human host by some schistosome species [13] or on those cysteine and aspartic peptidases that contribute to the digestion of the blood meal [14,15]. Among the latter, the digestive cathepsin B of *S*. *mansoni*, known as SmCB1, has been validated in a murine model of *S*. *mansoni* infection as a molecular target for therapy [9,16] and small molecule inhibitors of SmCB1 are under consideration as potential drug leads [16–19]. Other peptidase groups of schistosomes are less studied [12], including post-proline cleaving peptidases. This work focused on a *S*. *mansoni* prolyl oligopeptidase.

Prolyl oligopeptidases (POPs, also called prolyl endopeptidases) are approximately 70–80 kDa and belong to the S9 family of serine peptidases [20]. POPs cleave internal peptide bonds on the C-terminal side of proline residues and are found in plants, bacteria, fungi, protozoa, invertebrates and vertebrates [21]. For parasites, the best characterized POP is Tc80 in the infective trypomastigote stage of *Trypanosoma cruzi*, the causative agent of Chagas disease [22]. Tc80 seems to be involved in the parasite invasion as inhibition of Tc80 prevents parasite entry into host cells [23]. Accordingly, Tc80 is under investigation as a potential drug target [23,24].

In this report, we identified and functionally characterized the prolyl oligopeptidase from *S*. *mansoni* (SmPOP). We demonstrate that enzymatically active SmPOP is produced in several developmental stages and localized to the tegument and parenchyma of the parasite. We characterized in detail the biochemical activity of recombinant and native SmPOP, and designed nanomolar inhibitors of SmPOP that derange schistosomes maintained in culture. The data suggest that SmPOP is important to parasite survival and is, thus, a potential target for the development of anti-schistosomal therapeutics.

## Materials and Methods

### Ethics statement

All animal procedures were performed at the UCSF, USA, in accordance with protocol (AN107779–01) approved by the UCSF Institutional Animal Care and Use Committee (IACUC) as required by the Federal Animal Welfare Act and the National Institutes of Health Public Health Service Policy on Humane Care and Use of Laboratory Animals (http://grants.nih.gov/grants/olaw/references/phspol.htm).

### Schistosome material

*S*. *mansoni* (a Puerto Rican isolate) was kept in the University of California San Francisco (UCSF) laboratory by cycling between the intermediate snail host, *Biomphalaria glabrata*, and female golden Syrian hamsters (infected at 3–5 weeks old), as the definitive host. Hamsters are infected by subcutaneous injection of 800 cercariae and sacrificed 6–7 weeks post-infection by intra-peritoneal injection of pentobarbital (50 mg/kg). Adults, eggs and miracidia were then isolated as described previously [25,26]. Free-swimming cercariae were obtained from water containing infection-patent *Biomphalaria* to *‘*shed’ under a bright light. Cercariae were chilled over ice. Newly transformed schistosomula (NTS) were prepared by mechanically transforming cercariae [26,27] and cultivated in a Basch Medium 169 [28] containing 5% fetal calf serum, 100 units/mL penicillin and 100 μg/mL streptomycin for 5 days at 37°C under a 5% CO_2_ atmosphere. Daughter sporocysts were isolated by excision of hepato-pancreases from infected *B*. *glabrata* snails.

### Isolation of mRNA, cDNA synthesis and qRT-PCR

Adult worms, eggs, miracidia, daughter sporocysts, cercariae and NTS were collected, washed three times in 1.5 mL PBS, re-suspended in 500 μL Trizol reagent (Invitrogen) and processed as described previously [26]. Single-stranded cDNA was synthesized from total RNA by SuperScript III reverse transcriptase (Life Technologies) and an oligo dT_18_ primer. The final cDNA product was purified and stored at -20°C.

The gene expression profile of the SmPOP was assessed using reverse transcription- quantitative PCR (RT-qPCR). The following primers were used: forward 5'-CATTCGTGGTGGAGGAGAAT-3' and reverse 5'- CGCATACTGGAACTTGAGCA -3'. The primers were designed using the Primer 3 software (http://frodo.wi.mit.edu/) and their efficiency was evaluated as described previously [25,26]. The reactions, containing SYBR Green I Mastermix (Eurogentech), were prepared in a final volume of 25 μL in 96-well plates (Roche). The amplification profile consisted of an initial hot start (95°C for 10 min) followed by 40 cycles comprising 95°C for 30 s, 55°C for 60 s and 72°C for 60 s, and ending with a single cycle of 95°C for 60 s, 55°C for 30 s and 95°C for 30 s. The PCR reactions were performed in duplicate for each cDNA sample. At least one biological replicate, *i*.*e*., samples from a different RNA isolation, was performed. The analysis of the cycle threshold for each target was carried out as described [25,26] employing *S*. *mansoni* cytochrome c oxidase I (SmCOX I, GenBank AF216698) [29] as the sample-normalizing gene transcript. Transcript levels were expressed as log functions and as a percentage relative to that of SmCOX I in order to compare expression patterns.

### Expression and purification of recombinant SmPOP

The single gene encoding SmPOP (SchistoDB code: Smp_213240) was identified in the *S*. *mansoni* genome database [12] (*S*. *mansoni* GeneDB available at http://www.genedb.org/Homepage/Smansoni) via a protein BLAST search with the amino acid sequences of human and porcine prolyl oligopeptidases (GenBank accession numbers P48147 and P23687, respectively) as queries. The same search in the *S*. *japonicum* and *S*. *haematobium* genome databases [30,31] identified SmPOP orthologs with 88% and 95% identity, respectively (*S*. *japonicum*: GeneDB Sjp_0080730.1, GenBank AAX26405; *S*. *haematobium*: HelmDB Shae8836338, GenBank KGB33720).

The Champion pET directional expression kit (Life Technologies) was selected for expression of the SmPOP gene. The 2139 bp ORF was amplified using Phusion High-Fidelity DNA Polymerase (New England Biolabs) from adult schistosome cDNA using specific forward (5´-caccATGGAGCATACCAGTATCAACTATCC-3´) and reverse (5´-TTCTTTCCATGTGAGTGACATT-3´) primers. The PCR product was cloned into the expression vector pET101/D-TOPO (Invitrogen) and verified by DNA sequencing. Recombinant SmPOP (rSmPOP) with a C-terminal His_6_-tag was produced in *E*. *coli* BL21(DE3) by induction in LB broth medium containing 0.5 mM IPTG for 16 h at 16°C. Soluble rSmPOP was purified from the bacterial lysate using Ni^2+^ chelating chromatography (Hi-Trap IMAC FF column, GE Healthcare Life Sciences) under native conditions. The bound rSmPOP was eluted using a linear gradient of 0.01–0.5 M imidazole. The preparation was buffer-exchanged into 20 mM Tris-HCl, pH 8.0, using an Amicon Ultracel-30k ultrafiltration device (Millipore). rSmPOP was subsequently purified by FPLC over a Mono Q HR 5/5 column (GE Healthcare Life Sciences) equilibrated in 20 mM Tris-HCl, pH 8.0, and eluted using a linear gradient of 0–1 M NaCl in the same buffer. The purification process was monitored by a kinetic assay incorporating the peptidyl fluorogenic substrate, benzyloxycarbonyl (Z)-Gly-Pro-7-amino-4-methylcoumarin (AMC), and by SDS-PAGE. The preparation was concentrated and desalted into 20 mM Tris-HCl, pH 8.0, using an Amicon Ultracel-30k. The typical yield was approximately 3 mg of rSmPOP from 1 L of culture medium.

### Preparation of schistosome extracts

Soluble protein extracts (0.2–3 mg protein/mL) from *S*. *mansoni* adults, miracidia, cercariae, eggs and NTS were prepared by homogenization in 50 mM Tris-HCl, pH 8.0, containing 1% CHAPS, 1 mM EDTA, 1 μM pepstatin and 10 μM E-64 in an ice bath. The extracts were cleared by centrifugation (16000 *g* at 4°C for 10 min,), ultra-filtered using a 0.22 μm Ultrafree-MC device (Millipore) and stored at -80°C.

### Preparation of antibodies and immunoblotting

Specific polyclonal antibodies (Moravian Biotechnology) were generated in rabbits against the purified rSmPOP antigen using 50 μg of rSmPOP in Freund’s incomplete adjuvant and applied three times three weeks apart. IgG was isolated from the serum by affinity chromatography with a HiTrap Protein A column (GE Healthcare Life Sciences) according to the manufacturer’s protocol.

For immunoblotting, adult schistosome homogenate (30 μg protein) and rSmPOP (1 μg) were resolved by SDS-PAGE (15% polyacrylamide gel) under reducing conditions and transferred onto a PVDF membrane. The membrane was blocked 16 h in 10% non-fat milk in 50 mM Tris-HCl, pH 7.5, containing 150 mM NaCl and 0.1% Tween (TTBS). The membrane was then washed three times in TTBS and incubated for 1 h with anti-SmPOP polyclonal IgG diluted 1:1000 in TTBS. After washing in TTBS, the membrane was incubated for 1 h with goat horseradish peroxidase-conjugated anti-rabbit IgG (Sigma-Aldrich, catalog number A6154) at a dilution of 1:20000. After washing in TTBS, the membrane was developed with SuperSignal West Femto Chemiluminescent Substrate (Pierce) and imaged using an ImageQuant LAS 4000 biomolecular imager (GE Healthcare Life Sciences).

### Immunofluorescence microscopy

For sample preparation, adult *S*. *mansoni* worms were washed three times in PBS and fixed either in acetone (75% acetone in ethanol) at -20°C for 10 min or 4% formaldehyde in PBS at 25°C for 45 min. The samples were then rinsed with PBS and incubated in a 30% sucrose solution at 4°C for 16 h. The worms were placed in cryofixation molds and the sucrose solution was replaced with Optimal Cutting Temperature (OCT) medium (CellPath Ltd). The molds were placed over dry ice to freeze and the frozen blocks then stored at -80°C. The OCT-embedded worm samples were sectioned with a cryotome (Cryostat 2800 Frigocout, Cambridge Instruments). Sections of ~7 μm were air-dried and further processed for immunostaining.

Sections were rehydrated in PBS and fixed again either with formaldehyde or cold acetone as described above. The formaldehyde-fixed samples were further blocked in 100 mM glycine at 22°C for 20 min, followed by 2% BSA in PBS at 4°C for 16 h. Working solutions of primary and secondary antibodies were prepared in PBS containing 2% BSA; rabbit polyclonal anti-SmPOP IgG was diluted 1:900 and anti-rabbit IgG Alexa 594-conjugated secondary antibody (Molecular Probes) was diluted 1:200. The antibodies were incubated at 25°C on the sections for 45 min with three washes between the primary and secondary antibody incubations, and four washes after the secondary-antibody incubation (the fourth wash contained DAPI at 1 μg/mL for nuclear staining).

NTS samples were fixed in 4% formaldehyde in PBS at 4°C for 16 h. After fixation, they were washed 3 times in PBS at 25°C for 10 min and subsequently blocked with 100 mM glycine at 25°C for 20 min. Samples were permeabilized with 0.2% Triton X-100 in PBS for 40 min at 25°C and blocked with 2% BSA in PBS for 16 h at 4°C. The antibody diluent contained 0.1% Triton X-100, 0.1% BSA and 0.2% NaN_3_. Primary and secondary antibody solutions were incubated for 24 h with four washes of diluent over a 10 h period (the fourth wash contained DAPI at 1 μg/mL for nuclear staining).

Sections of adults and whole-worm preparations of NTS were embedded in Mowiol (Sigma-Aldrich) and visualized using a Leica SP2 AOBS confocal laser scanning microscope (Leica Microsystems) and a 20x oil immersion objective. Appropriate lighting settings were determined using control slides probed with preimmune serum to define the background signal threshold. Image stacks of optical sections were further processed using the Huygens deconvolution software package version 2.7 (Scientific Volume Imaging).

### Preparation of substrates and inhibitors

Fluorescence resonance energy transfer (FRET) substrates containing o-aminobenzoic acid (Abz) as the fluorescent group and p-nitro-phenylalanine (NPh) as the quencher acceptor were synthesized as peptidyl amides by Fmoc solid phase chemistry in an ABI 433A peptide synthesizer (Applied Biosystems) as described previously[16,32].

Substrates containing the fluorogenic group, 7-amino-4-carbamoylmethylcoumarin (ACC), were synthesized in the format Z-Xaa-Pro-ACC, with proteinogenic amino acids (except for cysteine) at the Xaa position, as described previously [33].

The inhibitors Z-Ala-Pro-chloromethyl ketone (CMK) and Z-Arg-Pro-CMK were prepared from the peptides Z-Ala-Pro-OH and Z-Arg(Pbf)-Pro-OH, respectively, according to the described procedure [34]. Z-Ala-Pro-OH and Z-Arg(Pbf)-Pro-OH were synthesized on solid phase using 2-chlorotritylchloride resin (Iris Biotech). Z-Xaa-Pro-CHO (CHO, aldehyde) inhibitors, where Xaa is Gly, Ala, Tyr, Arg or Lys, were synthesized on solid phase using H-Thr-Gly-NovaSyn TG resin (Merck) as described [35]. All of the substrates and inhibitors were purified by reverse-phase (RP)-HPLC over a C18 column using a TFA/acetonitrile system and characterized by electrospray ionization mass spectrometry on an LCQ Classic Finnigan Mat device (Thermo Finnigan).

The substrates Z-Gly-Pro-AMC, Succinyl (Suc)-Gly-Pro-Leu-Gly-Pro-AMC, Lys-Pro-AMC, Gly-Pro-AMC and Pro-AMC were purchased from Bachem. The POP inhibitors Y-29794 oxalate and Z-Pro-Pro-CHO were purchased from Santa Cruz Biotechnology, and SUAM 14746 from PeptaNova.

### Kinetic POP activity and inhibition assays

Assays were performed in triplicate in black, flat-bottomed, 96-well microplates (Nunc) in a total volume of 100 μL at 37°C. Z-Gly-Pro-AMC was used as substrate at a 50 μM final concentration. rSmPOP (50–100 ng), human POP (25–50 ng; Sigma-Aldrich, catalog number O9515) or schistosome homogenates (1–5 μg of protein) were pre-incubated for 10 min at 37°C in 80 μL of 0.1 M sodium phosphate, pH 8.0, containing 0.1% PEG 6000. Substrate (20 μL in the same buffer) was added to a final concentration of 50 μM. Hydrolytic activity was measured continuously in an Infinite M1000 microplate reader (Tecan) at the excitation and emission wavelengths of 360 and 465 nm, respectively. The pH profile of the activity was determined in 100 mM citrate phosphate (pH range 5.5–8.0), 100 mM Tris-HCl (pH range 8.0–9.0) and 100 mM sodium borate (pH range 9.0–10.0). For inhibition measurements, inhibitors were added to the 80 μL pre-incubation solution at a final concentrations of 0 to125 μM for 10 min and the reaction was initiated by the addition of the substrate. IC_50_ values were determined by nonlinear regression using the GraFit software (Erithacus Software). SmPOP activity in homogenates was measured in the presence of 10 μM E-64 to prevent undesired proteolysis by cysteine peptidases that contribute significant proteolytic activity in worm extracts [36]. POP activity was also measured with ACC and FRET substrates at excitation/emission wavelengths of 380/460 nm and 320/420 nm, respectively. Stock solutions of substrates and inhibitors (10 mM) were prepared in DMSO and the final assays concentration of DMSO was 1.5%.

### Interaction of rSmPOP with protein substrates

rSmPOP (0.7 μg) was incubated at 37°C for 16 h with 100 μg of human hemoglobin, human serum albumin, human collagens type I and IV (Sigma-Aldrich, catalog numbers H7379, A3782, C7774 and C7521 respectively) in 100 mM Tris-HCl, pH 8.0, in a final volume of 50 μL. After incubation, a 10 μL sample was resolved by 15% SDS-PAGE or Tricine-SDS-PAGE and stained with Coomassie Brilliant Blue G250.

### Hydrolysis of peptide hormones and neuropeptides by rSmPOP

The following synthetic analogues of human bioactive peptides were analyzed: angiotensin II (Sigma, catalog number A9525), angiotensin I, bradykinin, luteinizing-hormone-releasing hormone (LHRH), α-melanocyte-stimulating hormone (α-MSH), neurotensin, oxytocin, substance P, and vassopresin (all Bachem, catalog numbers H-1680, H-1970, H-6728, H-1075, H-4435, H-2510, H-1890 and H-1780, respectively). Stock solutions of peptides (10 mM) were prepared in water. rSmPOP (0.7 μg) was incubated at 37°C for 16 h with 25 nmol of peptide in 0.1 M Tris-HCl, pH 8.0, in a total volume of 50 μL. The reaction was stopped by adding TFA to a final concentration of 1%. The resulting fragments were purified by RP-HPLC over a C18 column (Vydac, 25 x 0.46 cm) using a TFA/acetonitrile system and characterized by electrospray ionization mass spectrometry on an LCQ Classic Finnigan Mat device (Thermo Finnigan).

### Hydrolysis of peptide hormones and neuropeptides by cultured schistosomes

Five adult schistosome pairs were placed into clear, 24-well, flat-bottom plates (Costar) containing 500 μL Basch Medium 199 [28], supplemented with 2.5% FBS, 100 units/mL penicillin and 100 μg/mL streptomycin. Human angiotensin I or bradykinin in 5 μL water was added to a final concentration of 100 μM and the incubation continued for 16 h at 37°C under a 5% CO_2_ atmosphere. In control experiments, the peptides were cultivated in the same system in the absence of schistosomes. After incubation, the samples were ZipTiped and the resulting fragments were analyzed using MALDI-TOF performed on an UltrafleXtreme (Bruker Daltonik) operated in reflectron mode with an acceleration voltage of 25 kV and a pulsed ion extraction of 120 ns. Desorption and ionization were achieved using a Smartbeam II laser. α-Cyano-4-hydroxycinnamic acid was used as a matrix. The data were acquired from m/z 220 to 3700 and analyzed with the FlexAnalysis 3.3 software (Bruker Daltonik). The mass spectra were externally calibrated using a Peptide Calibration Standard I (Bruker Daltonik) and averaged from 3000 laser shots.

### Fluorescence SmPOP activity assay with cultured schistosomes

Adult worms (3 pairs) or approximately 150 NTS were incubated at 37°C and 5% CO_2_ for 2 days in 200 μL of Basch Medium 169 containing 5% FBS, 100 units/mL of penicillin and 100 μg/mL using black clear bottomed 96-well microplates (Costar). After incubation, half of the medium (100 μl) was transferred to an empty well leaving the parasites in the remaining half. Then SmPOP activity was measured in both wells upon the addition of 20 μL of Z-Gly-Pro-AMC (prepared as a 250 μM stock in Basch Medium 169) and in the presence or absence of 1 μM of the POP inhibitor, Z-Ala-Pro-CMK. Controls contained medium alone.

### Molecular modeling of SmPOP

A spatial model of SmPOP was constructed by homology modeling as described previously [17]. Briefly, the X-ray structure of porcine POP in complex with the inhibitor Z-Pro-Pro-CHO (PDB entry: 1QFS) was used as a template. The homology module of the MOE program (Chemical Computing Group) was used for the modeling of the SmPOP structure. The inhibitor conformation was refined by applying the LigX module of the MOE for the optimization procedure and its final binding mode was selected by the best-fit model based on the London dG scoring function and the generalized Born method [37]. Molecular images were generated with UCSF Chimera (http://www.cgl.ucsf.edu/chimera/).

### Parasite assay and phenotype scoring

NTS (200–300 parasites) were incubated in 200 μL of Basch Medium 169 and supplements, as described above. Inhibitors were added at final concentrations of 1 or 10 μM (0.5% DMSO final) and the incubations continued for 4 days. Grading of phenotypic responses arising as a function of time and concentration was modified after Jilkova *et al*. [16]: Grade I, dead NTS by 2 days of culture at 10 μM and dying/dead NTS by 3 days at 1 μM. Grade II, dead NTS by 3 days at 10 μM and round/dark/dying by 3 days at 1 μM; Grade III, round/dark by 3 days at 1 and 10 μM concentrations (S1 Fig). ‘Dead’ was adjudicated as the loss of normal shape and the lack of movement often accompanied by obvious internal disruptions. ‘Dying’ was similar to death except that movement was detectable. Otherwise, the terms ‘round/dark’ were used to indicate less severe but obvious changes in the parasites relative to DMSO controls.

## Results

### SmPOP is homologous to prolyl oligopeptidases from various parasites

A protein BLAST analysis of the *S*. *mansoni* genome database [12,38] using mammalian prolyl oligopeptidases as queries identified a gene ortholog (SmPOP), Smp_213240, located on the sex-determining Z/W chromosomal pair. SmPOP cDNA was cloned, sequenced, and the sequence was deposited into the GenBank as KF956809. The blast analysis did not identify other gene isoforms. The SmPOP open reading frame consists of 2,139 bp that encodes a protein of 712 amino acid residues with a calculated molecular mass of 82 kDa. No signal/leader peptide was predicted for the sequence. SmPOP has about 50% identity with human and porcine POPs (S1 Table) and belongs to the S9 family of serine peptidases (S2 Fig). SmPOP has the characteristic domain composition of mammalian POPs, consisting of N-terminal, β-propeller and peptidase domains. The peptidase domain of SmPOP has a catalytic triad in the order of Ser556, Asp643 and His682, which is typical of POPs and other S9 family peptidases [39]. In addition, the regions surrounding the catalytic-triad residues have the most notable sequence identity. A phylogenetic tree constructed for prolyl oligopeptidases of animal, plant, protozoan and bacterial origin (S3 Fig) demonstrates that SmPOP clusters with other trematode and nematode POPs. This monophyletic group is well separated from other clades.

### *S*. *mansoni* developmental stages express active POP

**Fig 1:**
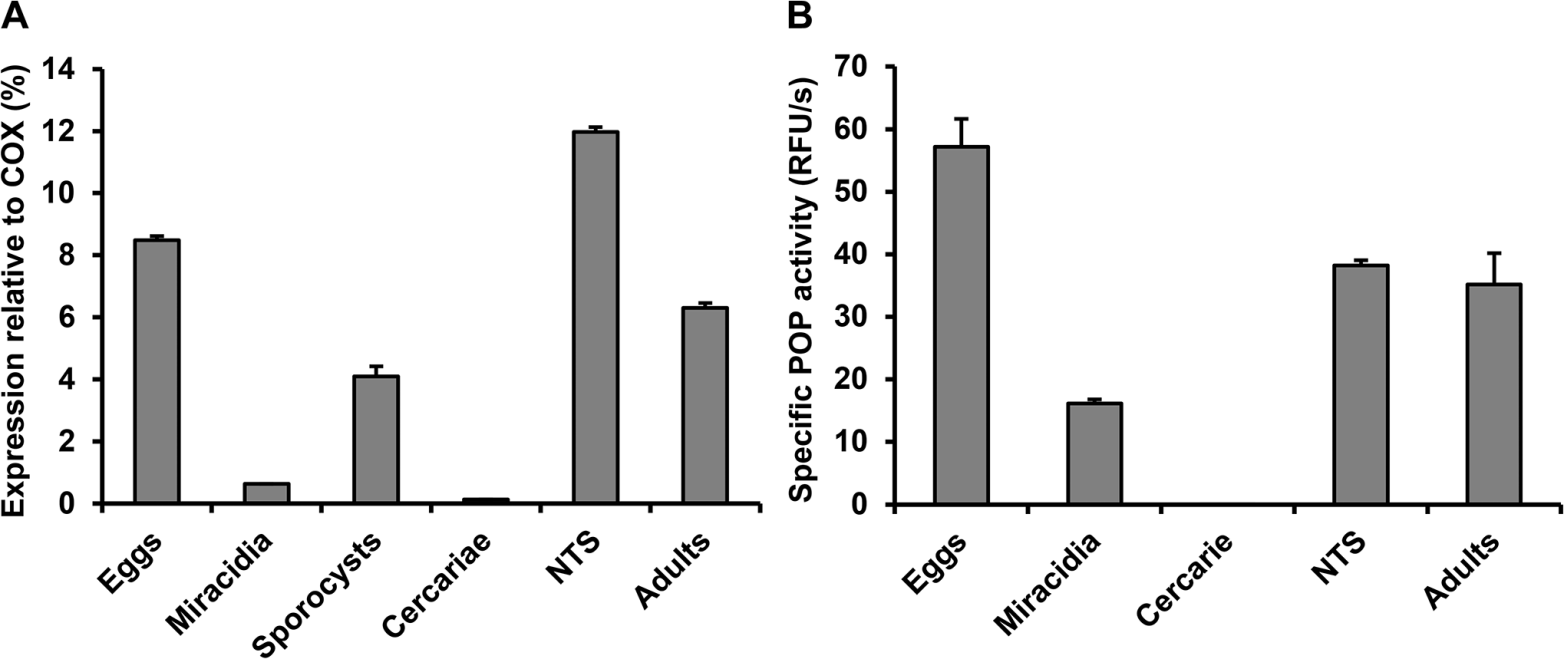
Activity and transcriptional profiling of SmPOP in the developmental stages of *S*. *mansoni*. (A) The expression of SmPOP was evaluated by quantitative RT-PCR. mRNA transcriptional levels are presented as the percentage of expression relative to the constitutively expressed *S*. *mansoni* cytochrome oxidase I (SmCOX I). The mean values ± S.D. of three replicates are given. (B) SmPOP activities were measured in protein extracts of the developmental stages (except sporocysts not available in sufficient amount and purity) using a kinetic assay with the fluorogenic substrate Z-Gly-Pro-AMC at pH 8.0. POP activities (sensitive to inhibition by the specific POP inhibitor Z-Pro-Pro-CHO) are expressed in relative fluorescence units (RFU/s) and normalized to protein content.

Messenger RNA transcript levels for SmPOP were evaluated in eggs, miracidia, daughter sporocysts, cercariae, NTS and adults using qRT-PCR (Fig 1A). The expression of SmPOP was recorded in eggs, daughter sporocysts, NTS and adult schistosomes (in the range of 4–12% of the expression of the validated reference gene, SmCOX I [26]). In miracidia and cercariae, expression was below 1% of the SmCOX I level (Fig 1A).

At the protein level, SmPOP enzymatic activity in soluble extracts of various developmental stages was determined in a kinetic assay using the fluorogenic substrate, Z-Gly-Pro-AMC, which is specific for prolyl oligopeptidases. The measured activities were further authenticated as being due to a prolyl oligopeptidase by their sensitivity to Z-Pro-Pro-CHO, a selective small-molecule inhibitor of prolyl oligopeptidases [40]. Prominent SmPOP activity was measured in the homogenates of eggs, NTS and adults, whereas weak activity was measured in miracidial homogenates; no activity was detected in cercariae (Fig 1B).

Overall, active SmPOP is expressed in the *S*. *mansoni* developmental stages that live in the human host and the activity profile is consistent with that for mRNA expression. In addition, the presence of SmPOP was confirmed in the protein homogenate of adult *S*. *mansoni* by mass spectrometry proteomics (S2 Table).

### SmPOP cleaves proline-containing neuropeptides and oligopeptide hormones of the host

**Fig 2:**
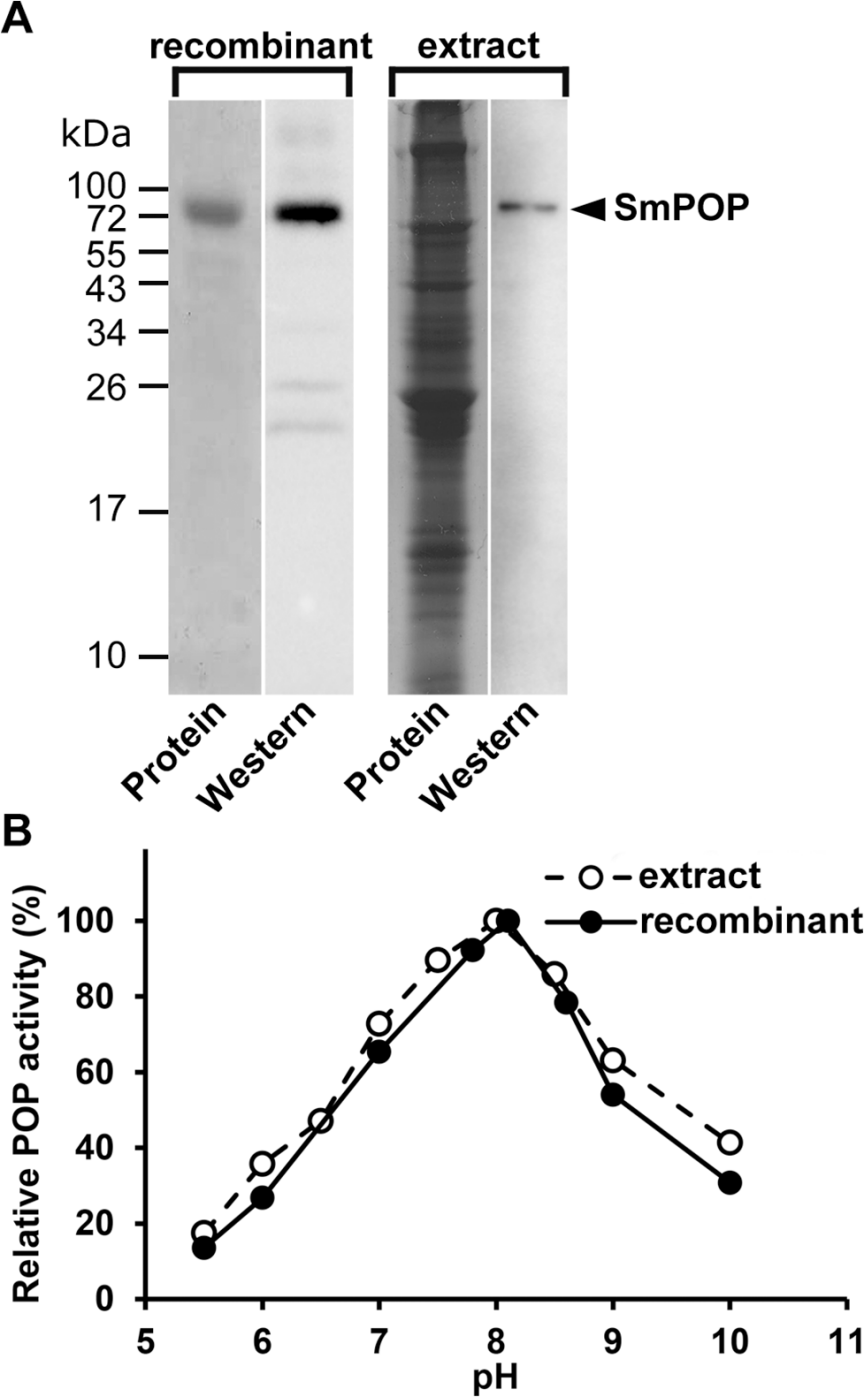
A comparison of recombinant SmPOP and native SmPOP. (A) Recombinant SmPOP expressed in *E*. *coli* (two left lanes) and *S*. *mansoni* protein extract (two right lanes) were resolved by SDS-PAGE, blotted onto a membrane, and visualized by protein staining or by anti-SmPOP IgG (polyclonal antibodies raised against recombinant SmPOP). (B) The pH profile of recombinant SmPOP and native SmPOP (in *S*. *mansoni* extracts). Activity was measured in a kinetic assay with the fluorogenic substrate Z-Gly-Pro-AMC. Mean values, expressed as a percentage are shown (the S.D. values of three replicates are within 10% of the mean).

Recombinant SmPOP (rSmPOP) was expressed in *E*. *coli* as a soluble and catalytically active enzyme. rSmPOP was purified to homogeneity by a combination of metal-affinity chromatography and ion-exchange chromatography, and subsequently migrated on SDS-PAGE as a single band of approximately 80 kDa (Fig 2A). Rabbit polyclonal antibodies raised against rSmPOP reacted with the original rSmPOP antigen by immunoblotting and recognized a single band in the homogenates from schistosome adults (Fig 2A). The molecular mass of both the native SmPOP and rSmPOP is in good agreement with the theoretical mass of SmPOP predicted from the amino-acid sequence (82 kDa).

The pH activity profile of rSmPOP was determined using the fluorogenic substrate Z-Gly-Pro-AMC and compared with that of the native SmPOP in schistosome adult homogenates (Fig 2B). For both protein sources, the substrate was cleaved between pH 6.0 and 10.0 with optimal activity around pH 8.0. No POP activity was detected below pH 5.0.

**Fig 3:**
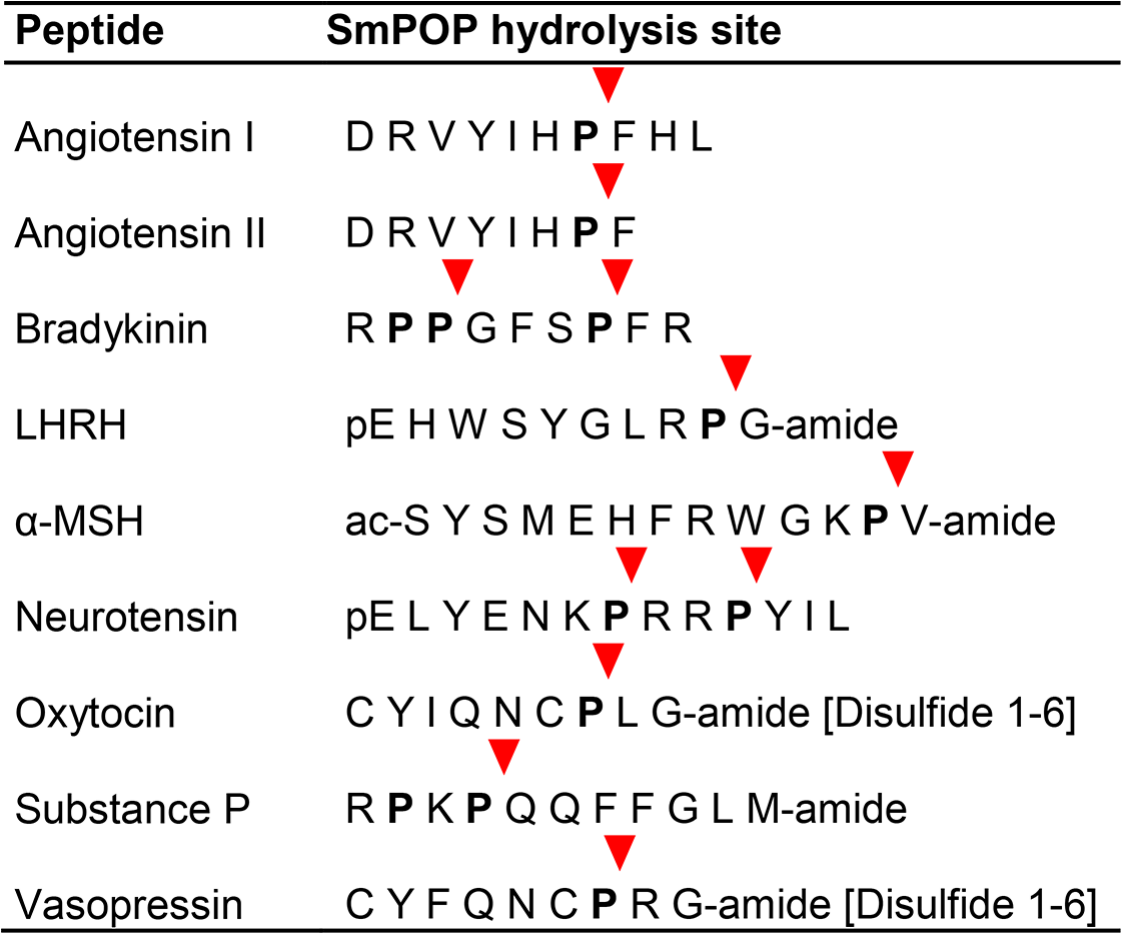
SmPOP cleaves human, proline–containing peptide hormones and neuropeptides. The peptides were incubated with recombinant SmPOP at pH 8.0 and the cleavage positions (the red triangles) identified by mass spectrometry. Proline residues are indicated in bold; the disulfide connectivity is indicated in parentheses.

Prolyl oligopeptidases perform specific post-proline cleavages of various peptides [39,41]. Accordingly, using a broad panel of proline-containing bioactive peptides, we asked whether SmPOP cleaves human peptide hormones and neuropeptides (Fig 3). After incubation of the tested peptides with SmPOP, the resulting fragments were separated by HPLC and the cleavage positions identified by mass spectrometry. All substrates were cleaved specifically at the carboxyl terminus of proline residues with the exception of the Pro-Lys bond in Substance P and the Pro-Pro bond in bradykinin (Fig 3). The substrate specificity resembles that of mammalian prolyl oligopeptidases, which cleave a Pro-Xaa bond in peptides, where Xaa is not a Pro residue. Also, like mammalian prolyl oligopeptidases, SmPOP does not cleave after a penultimate N-terminal Pro residue [42].

The activity of rSmPOP towards host-derived macromolecular substrates was tested with several human proteins, including hemoglobin, serum albumin and collagens I and IV. No hydrolysis was observed even after prolonged incubation (S4 Fig), indicating that SmPOP is a true oligopeptidase with an action restricted to oligopeptide substrates.

**Fig 4:**
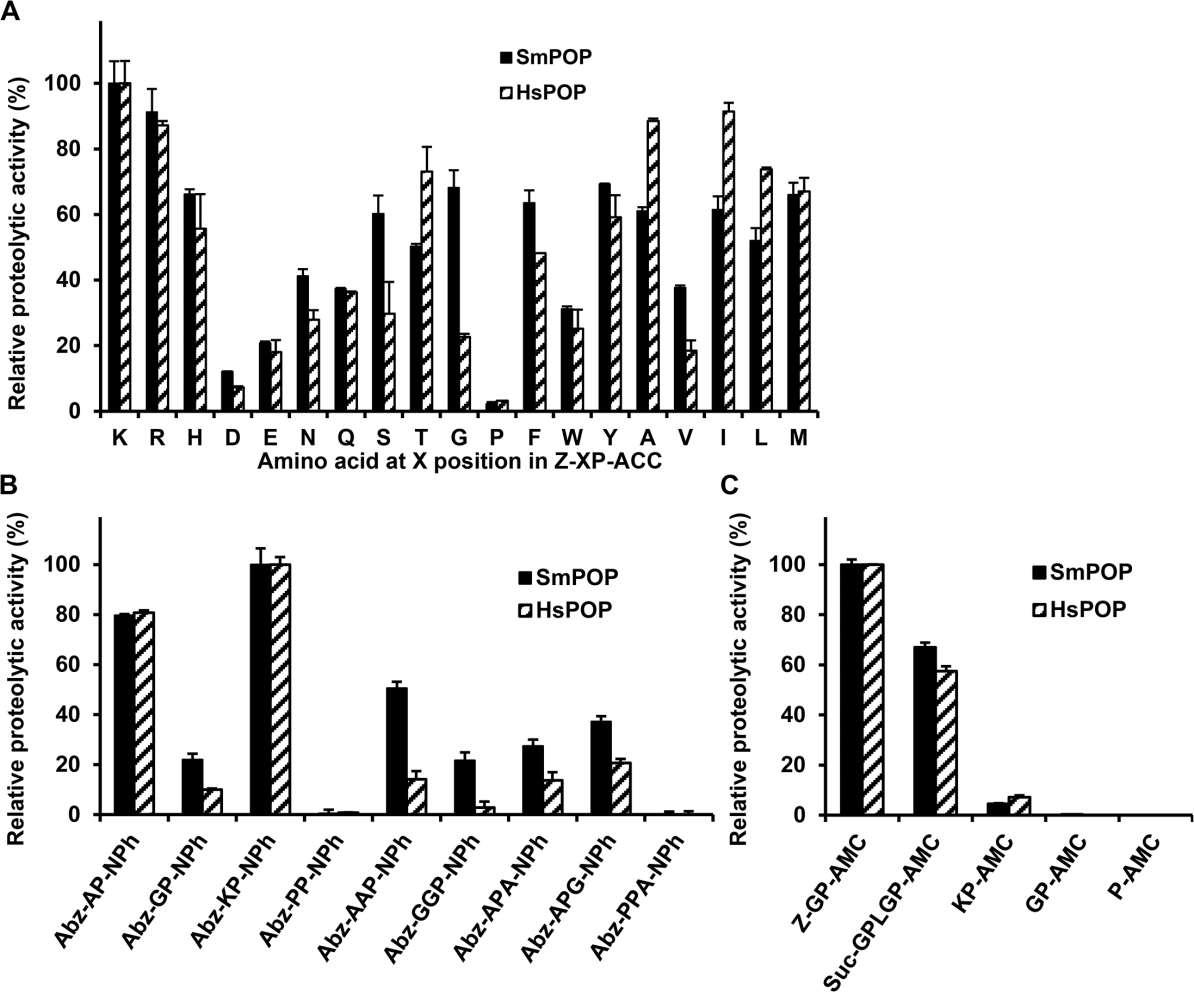
Substrate specificity of recombinant SmPOP. The peptidolytic activity of SmPOP was probed using a series of synthetic substrate libraries: (A) fluorogenic substrates Z-XP-ACC with proline in the P1 position and the indicated amino acids in the P2 position; (B) FRET-based peptide substrates with-XP- (in the P2 and P1 positions) extended to occupy the P3 and P1' positions; (C) fluorogenic substrates with proline in the P1 position, which w used to assay the following peptidases: collagenase-like peptidases (Suc-GPLGP-AMC), dipeptidyl aminopeptidase II (KP-AMC), dipeptidyl aminopeptidase IV (GP-AMC) and prolyl aminopeptidase (P-AMC). The substrate hydrolysis was measured in a kinetic assay at pH 8.0 using recombinant SmPOP or human POP (HsPOP). The mean values ± S.D. of three replicates are normalized to the maximum value in each series.

A fluorogenic substrate library was used to determine the SmPOP cleavage specificity at the substrate P2 position (Fig 4A). The greatest preference was recorded for basic residues (Arg and Lys), but a variety of other amino acid residues was also acceptable at this position, including hydrophobic, aliphatic and polar residues. Substrates with acidic residues and Pro at P2 were least preferred.

The substrate specificity of rSmPOP was further investigated using FRET synthetic substrates which had been designed based on the aminobenzoyl (Abz)-nitrophenylalanine (NPh) donor–acceptor pair and contained a Pro residue at P1 (Fig 4B). We prepared a set of substrates with variations in the P2 position (Abz-Ala-Pro-NPh, Abz-Gly-Pro-NPh, Abz-Lys-Pro-NPh, and Abz-Pro-Pro-NPh) and which were lengthened to include the P3 (Abz-Ala-Ala-Pro-NPh and Abz-Gly-Gly-Pro-NPh) or P1’ positions (Abz-Ala-Pro-Ala-NPh and Abz-Ala-Pro-Gly-NPh). The greatest rSmPOP activity was measured with the substrates Abz-Ala-Pro-NPh and Abz-Lys-Pro-NPh, whereas the substrate Abz-Pro-Pro-NPh was not digested; increasing the substrate length to P3 and P1’ positions did not increase its affinity (Fig 4B).

Finally, rSmPOP was tested for its ability to hydrolyze substrates with Pro in the P1 position that allows for cleavage by other post-proline cleaving enzymes, including collagenase-like peptidases (Suc-Gly-Pro-Leu-Gly-Pro-AMC), dipeptidyl aminopeptidase II (Lys-Pro-AMC), dipeptidyl aminopeptidase IV (Gly-Pro-AMC) and prolyl aminopeptidase (Pro-AMC; Fig 4C). Only Suc-Gly-Pro-Leu-Gly-Pro-AMC, suitable for the endopeptidase mode of cleavage, was digested by rSmPOP with the same efficiency as found for the classical and minimal POP substrate, Z-Gly-Pro-AMC. The cleavage of exopeptidase substrates with free N-termini occurs only very slowly (Lys-Pro-AMC) or not at all (Gly-Pro-AMC and Pro-AMC).

To summarize, SmPOP is a true oligopeptidase that hydrolyzes peptide but not protein substrates in the endopeptidase mode with a strict specificity for Pro at P1.

### Specificity of SmPOP inhibition and the design of specific inhibitors

The general inhibition specificity of rSmPOP was analyzed using a panel of peptidase class/type-selective small-molecule inhibitors as listed in Table 1. rSmPOP activity was completely inhibited by selective prolyl-oligopeptidase inhibitors with chloromethyl (CMK) and aldehyde (CHO) warheads (Z-Ala-Pro-CMK and Z-Pro-CHO), and by the general serine peptidase inhibitor, diisopropyl fluorophosphate. Partial inhibition was observed with Pefabloc SC, PMSF (phenylmethylsulfonyl fluoride), TLCK (Nα-tosyl-L-lysine chloromethyl ketone), TPCK (N-p-tosyl-L-phenylalanine chloromethyl ketone) and 3,4-dichloroisocoumarin, all of which target the serine peptidases of the chymotrypsin S1 family. SmPOP activity was neither affected by protein inhibitors of serine peptidases (soybean trypsin inhibitor (STI) and bovine pancreatic trypsin inhibitor (BPTI)) nor by the inhibitors of cysteine, aspartic and metallo-peptidases. This overall inhibition profile shows that SmPOP has the ligand-binding characteristics analogous to those of mammalian POPs [41,42].

**Table 1:** Inhibition of recombinant SmPOP by protease inhibitors.

Inhibitor^a^	Target protease^b^	Concentration (μM)	Inhibition (%)^c^
Pefabloc SC	SP	1000	12.0±3.1
PMSF	SP	1000	47.6±1.6
Benzamidine	SP (trypsin type)	10	3.7±1.1
TLCK	SP (trypsin type)	1	38.3±1.2
TPCK	SP (chymotrypsin type)	1	67.2±6.2
3,4-dichloroisocoumarin	SP	100	77.3±0.6
BPTI (Aprotinin)	SP	50	1.4±1.1
STI	SP	10	12.3±3.2
Diisopropyl fluorophosphate	SP	100	100±1
Leupeptin	SP, CP	20	2.3±1.2
Antipain	SP, CP	20	32.4±1.4
E64	CP	10	6.5±6.1
Pepstatin A	AP	1	7.3±3.5
EDTA	MP	1000	3.8±2.3
Bestatin	MP (leucin aminopeptidase)	1	2.3±2.1
Z-Ala-Pro-CMK^d^	SP (prolyl oligopeptidase)	1	100±3
Z-Pro-Pro-CHO^d^	SP (prolyl oligopeptidase)	1	100±1
Z-Pro-Pro-OH^d^	SP (prolyl oligopeptidase)	100	37.6±2.1
Z-Pro-OH^d^	SP (prolidase)	100	41.1±1.8

^a^ Abbreviations: PMSF (phenylmethylsulfonyl fluoride), TLCK (Nα-Tosyl-L-lysine chloromethyl ketone), TPCK (N-p-Tosyl-L-phenylalanine chloromethyl ketone), BPTI (bovine pancreatic trypsin inhibitor), STI (soybean trypsin inhibitor), E64 (trans-Epoxysuccinyl-L-leucylamido(4-guanidino)butane)

^b^ The target proteases are classified based on catalytic type into aspartic (AP), cysteine (CP) and serine (SP) proteases, and metalloproteases (MP).

^c^ The recombinant SmPOP was pre-incubated with the given inhibitor and remaining activity was measured in a kinetic assay with the fluorogenic substrate Z-Gly-Pro-AMC. The mean values ± S.D. of three replicates are expressed as percentage inhibition relative to the uninhibited control.

^d^ CMK: chloromethyl ketone; CHO: aldehyde; OH: free carboxyl.

A more detailed inhibitor specificity profile for rSmPOP was investigated using a panel of synthetic peptidic inhibitors with the structure Z-Xaa-Pro-CHO/CMK, which included aldehyde (CHO) or chloromethylketone (CMK) reactive warheads (Table 2). The amino-acid residues for the Xaa position were selected based on the S2 substrate specificity of rSmPOP (Fig 4A). Table 2 shows that the synthesized aldehyde derivatives inhibit SmPOP with IC_50_ values in the low micromolar concentration range (1.3 to 6.1 μM); the inhibitory specificity at the binding subsite S2 corresponds to the substrate specificity profile (Fig 4A) and shows that inhibitors with the basic amino acids in the P2 have position have the lowest IC_50_ values.

**Table 2:** Inhibition of SmPOP activity and anti-schistosomal effect of synthetic SmPOP inhibitors.

Inhibitor	IC_50_ (μM)^a^		Severity of phenotype against parasite^b^
	SmPOP	HsPOP	Grade
Y-29794 oxalate^c^	8.6±0.4	0.49±0.03	II
SUAM 14746^d^	0.092±0.005	0.083±0.007	no effect
Z-Pro-Pro-CHO^e^	0.16±0.03	0.012±0.005	no effect
Z-Ala-Pro-CHO^e^	3.1±0.2	6.1±0.3	III
Z-Gly-Pro-CHO^e^	6.1±0.4	7.6±0.9	II
Z-Tyr-Pro-CHO^e^	4.4±0.4	11.4±0.7	II
Z-Arg-Pro-CHO^e^	1.3±0.3	2.4±0.2	II
Z-Lys-Pro-CHO^e^	3.0±0.6	7.2±0.6	I
Z-Ala-Pro-CMK^e^	0.0032±0.0004	0.0168±0.0046	II
Z-Arg-Pro-CMK^e^	0.0029±0.0001	0.0048±0.0006	III

^a^ The IC_50_ values were determined in a kinetic activity assay with recombinant SmPOP or HsPOP and the fluorogenic substrate Z-Gly-Pro-AMC at pH 8.0. The mean values ± S.D. of three replicates are given.

^b^ Induction of phenotype alterations by the inhibitors was determined with NTS in culture. The inhibitors were tested at 10 μM and 1 μM concentrations, and the resulting phenotypes, arising as a function of time and concentration, were graded I to III, with grade I being the most severe (see Materials and Methods).

^c^ Y-29794 oxalate: 2-[[8-(Dimethylamino)octyl]thio]-6-(1-methylethyl)-3-pyridinyl-2-thienylmethanone oxalate

^d^ SUAM 14746: 3-([4-[2-(E)-styrylphenoxy]butanoyl]-L-4-hydroxyprolyl)thiazolidine).

^e^ Peptidic inhibitors with reactive aldehyde (CHO) or chloromethyl ketone (CMK) warheads (see Materials and Methods).

The introduction of an irreversible covalent CMK warhead to the inhibitor scaffold improved the IC_50_ value by three orders of magnitude (IC_50_ from 2.9 to 3.2 nM) in comparison with inhibitors containing reversible covalent CHO warhead (Table 2). Furthermore, we tested the sensitivity of rSmPOP to three commercially available inhibitors developed for human POP, namely Y-29794 oxalate [43], SUAM 14746 [44], and Z-Pro-Pro-CHO [40]. Whereas the inhibition by SUAM 14746 was similar for both the human and schistosomal enzymes (IC_50_ values of 83 nM and 92 nM, respectively), Y-29794 oxalate and Z-Pro-Pro-CHO inhibited SmPOP with IC_50_ values that were about one order of magnitude greater than those for human POP (IC_50_ values of 8.6 μM and 0.49 μM, respectively, for Y-29794 oxalate, and 0.16 μM and 0.01 μM, respectively, for Z-Pro-Pro-CHO).

**Fig 5:**
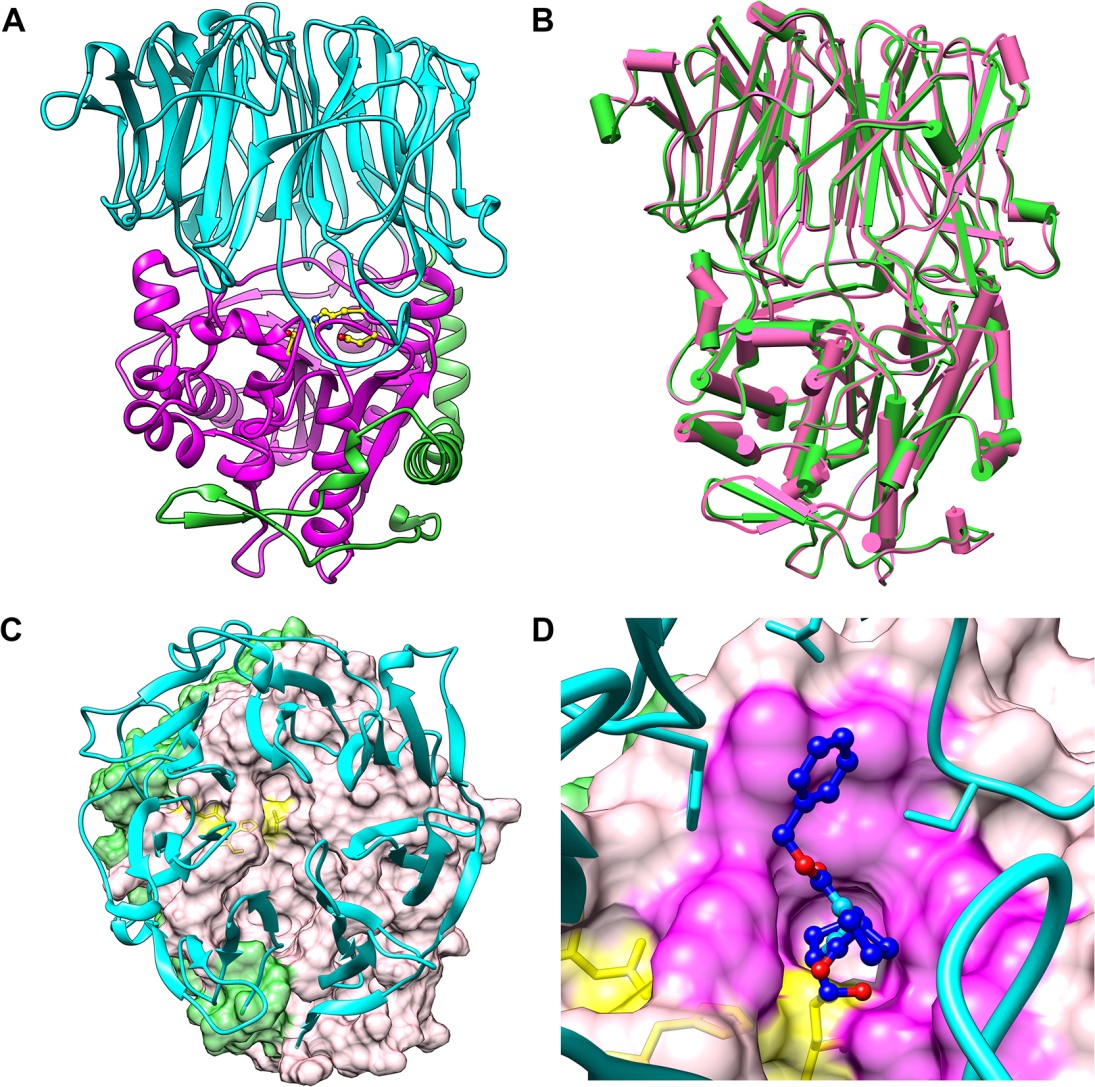
A three-dimensional homology model of SmPOP. (A) A ribbon representation of the overall SmPOP structure showing the β-propeller domain (cyan) and the catalytic domain (pink); the N-terminal segment is colored green. The active site containing the catalytic triad Ser556, Asp643 and His682 (yellow) is located at the interface of the two domains. (B) A superposition of the SmPOP model (green) and the porcine POP crystal structure (pink with the PDB code 1QFS) in a cylinder representation. (C) A view from the top of the SmPOP model (the β-propeller domain (cyan, ribbon representation) controls access to the active site of the catalytic domain (the pink surface) indicated by the catalytic triad residues (the yellow surface and sticks); the N-terminal segment is shown as the green surface. (D) A surface representation of the SmPOP active site located in the catalytic domain (the pink surface). The covalently-bound inhibitor Z-Pro-Pro-CHO is depicted in the ball-and-stick representation (carbon atoms in blue, oxygen in red and nitrogen in light blue). The catalytic-domain residues forming contacts with the inhibitor are highlighted as the magenta surface; the catalytic triad residues are represented by the yellow surface/sticks. The β-propeller domain is shown as a cyan ribbon, the residues interacting with the inhibitor as cyan sticks.

A spatial model of SmPOP was constructed by homology modeling to study the structure-activity/inhibition relationship. The X-ray structure of porcine POP (PDB code 1QFS) was used as a template. Fig 5 shows that SmPOP has the conserved architecture of the mammalian POP comprising both the β-propeller and peptidase domains [45]. The peptidase domain (residues 430–712) has a characteristic α/β-hydrolase fold [46,47] which consists of a central eight-stranded β-sheet flanked on both sides by eight α helices. The catalytic amino-acid residues Ser556, Asp643 and His682 are located in a large cavity at the interface between the domains. The disk-shaped β-propeller domain (residues 76–429) is composed of seven repeats of four-stranded antiparallel β-sheets which are arranged around a central tunnel.

The binding mode of SmPOP was analyzed using the transition-state analog POP inhibitor Z-Pro-Pro-CHO (benzyloxycarbonyl-L-prolyl-L-prolinal) which was docked into the SmPOP active site based on the crystallographic complex of this inhibitor with porcine POP (PDB code 1QFS). The docking model (Fig 5) shows that the prolinal residue of the inhibitor forms a covalent hemi-acetal linkage with the catalytic Ser556. The P1 Pro ring binds to the hydrophobic S1 binding pocket (defined by Phe478, Trp597, Tyr601 and Val646 residues) and is stacked against a Trp597 side chain. The backbone of both the P1 and P2 proline residues forms three hydrogen bonds to the SmPOP active site. Additionally, the P3 benzyloxycarbonyl group binds to the hydrophobic S3 binding site (residues Phe175, Cys257, Asn273, Ile593 and Ala596).

### SmPOP is localized in the tegument and parenchyma of adult schistosomes

**Fig 6:**
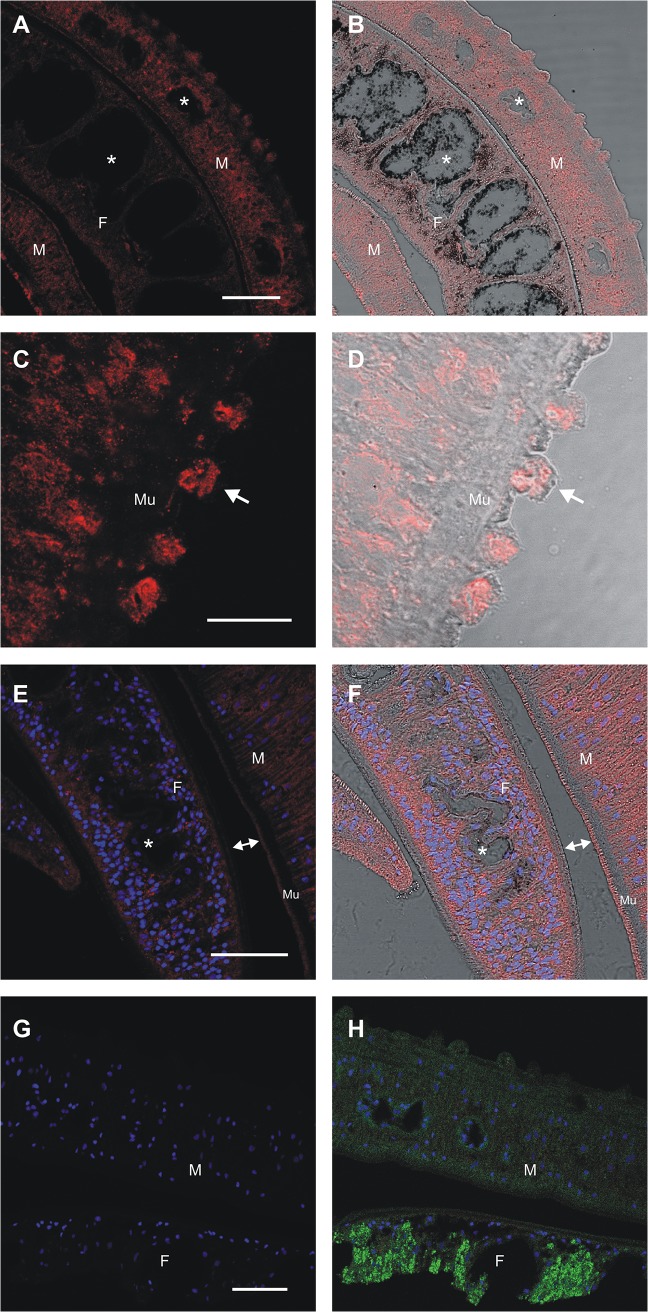
SmPOP is localized to the tegument and parenchyma of adult *S*. *mansoni*. Semi-thin sections of adult male and female *S*. *mansoni* were probed with an anti-SmPOP IgG (A–F) or a pre-immune IgG (G, H) followed by reaction with an anti-rabbit IgG Alexa 594-labeled secondary antibody (red). DAPI was used to label the nuclear DNA (blue); female vitellaria are characterized by strong autofluorescence in the green spectrum (H). The left column shows merged fluorescent channels; in the right column, the signal is merged with differential interference contrast (except in H). Male worms (M) incubated with anti-SmPOP show a stronger immune-reactivity than female worms (F) (micrographs A and B). A red fluorescent signal is found in the parenchyma and tegument, but it is absent from the gut (the asterisks in A, B, E and F) and muscular tissue (Mu, micrographs C-F). In male worms the signal is found accumulated in the tubercles of the dorsal tegument (the arrows in C and D) and also outlines the gynaecophoral canal. Note the difference in signal intensity between the male and female tegument (the connected arrowheads in E and F). Only faint background fluorescence could be detected in the red spectrum in the negative control probed with pre-immune IgG (the micrographs G and H). The scale bar in C and D represents 20 μm; in A, B, E–H, 50 μm.

**Fig 7:**
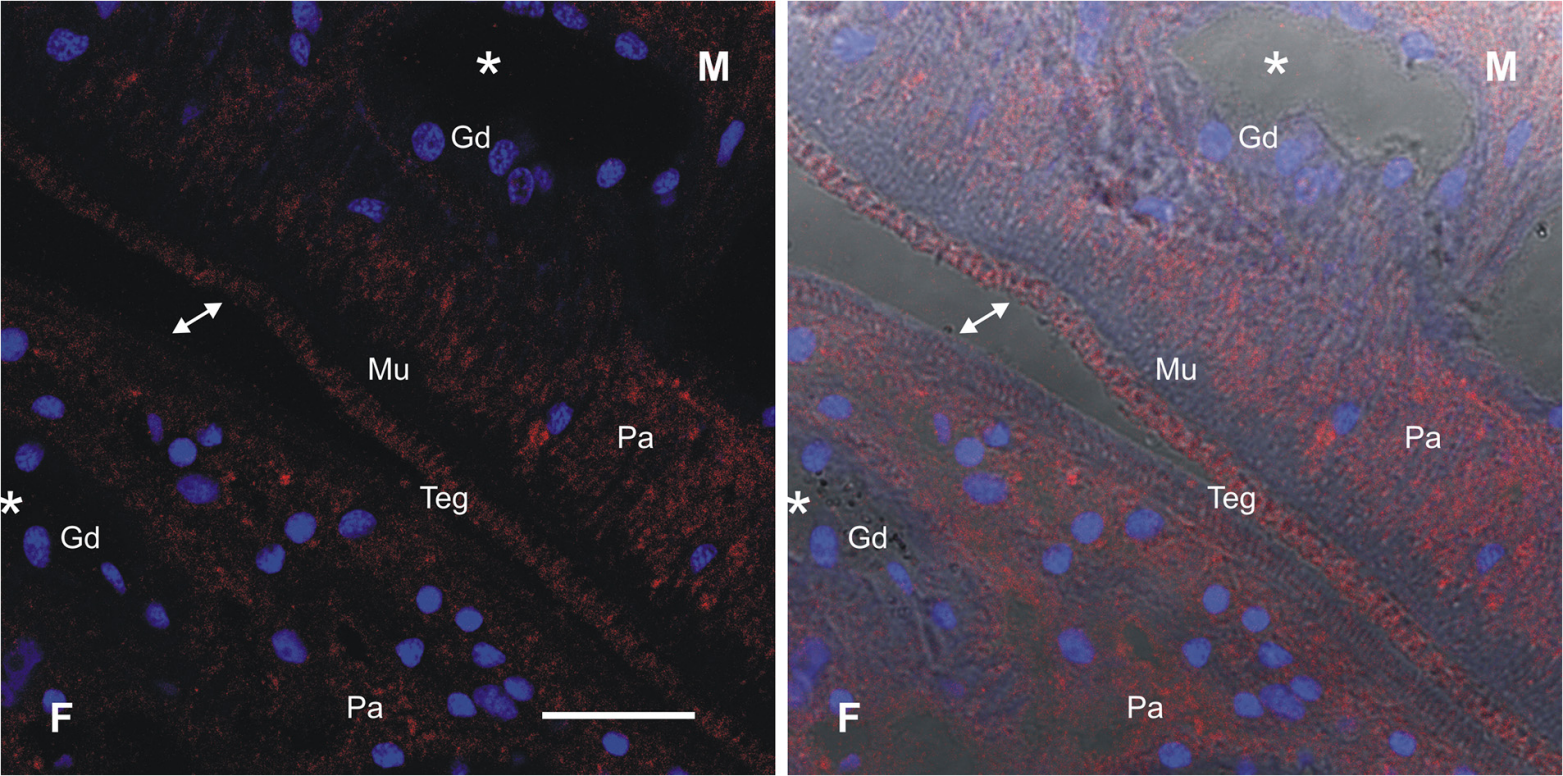
Detailed micrograph of SmPOP localization in the tegument of adult *S*. *mansoni*. The tissue section was probed with anti-SmPOP IgG followed by an anti-rabbit IgG Alexa 594-labeled secondary antibody (red). DAPI was used to label the nuclear DNA (blue). The left image shows merged fluorescent channels; on the right, the fluorescent signal is merged with differential interference contrast imaging. A red fluorescent signal is found in the parenchyma (Pa) and tegument (Teg), but is absent from the gastrodermis (Gd), gut lumen (the asterisks) and muscular tissue (Mu). Male worms (M) show a stronger immuno-reactivity than female worms (F). Note the difference in the signal intensity on the tegument of the male compared to the female (the connected arrowheads). Scale bar = 20 μm.

Indirect immunofluorescence microscopy on semi-thin sections using affinity-purified antibodies against rSmPOP demonstrate that SmPOP is expressed in the parenchyma and tegument of adult schistosomes (Fig 6; for a high-resolution micrograph, see Fig 7). The intensity of the signal was greater in the tegument of the male compared to the female (Fig 6C and 6D). Labeling was not observed in the gastrodermis, gut lumen and muscular tissues (Fig 7). Intense staining was seen in the male tegumental tubercles (Fig 6C and 6D). Pre-immune serum was applied as a negative control and only faint background fluorescence was detected (Fig 6G and 6H). Similar results were obtained in immuno-histochemical studies with NTS (S5 Fig). With this developmental stage, SmPOP was localized at or close to the surface; a low diffuse signal was also seen in the parenchyma whereas the gut exhibited no specific fluorescence. No reaction was observed with pre-immune serum (S5 Fig).

### SmPOP on living parasites cleave host peptides containing proline

**Fig 8:**
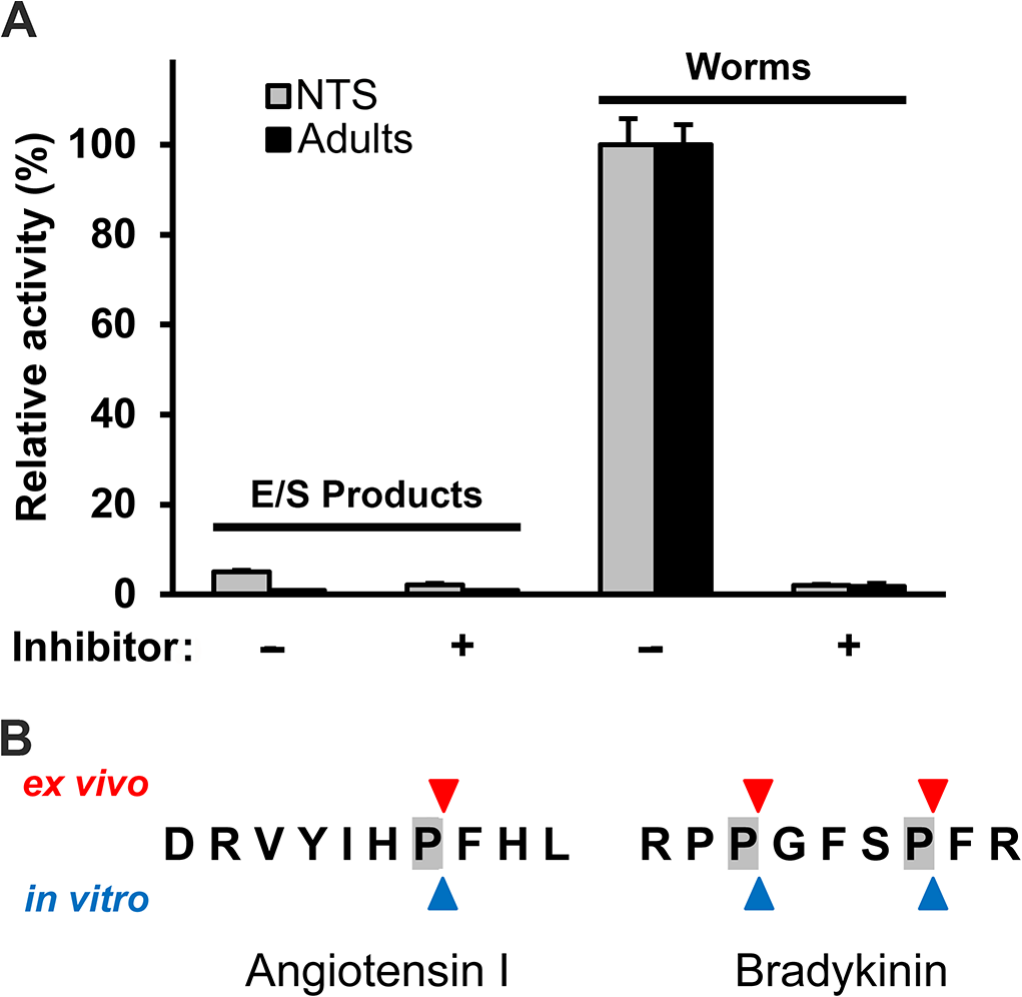
SmPOP in live *S*. *mansoni* cleaves vasoregulatory hormones. (A) SmPOP activity detected in the excretory-secretory products of or associated with live NTS and adults (worms) maintained in culture was determined using the fluorogenic substrate Z-GP-AMC. The inhibitor, Z-Ala-Pro-CMK, was added in the control experiments to specifically block SmPOP activity. The mean values ± S`.D. of three replicates are normalized to the maximum value in each series. (B) The peptide hormones angiotensin I and bradykinin were incubated with recombinant SmPOP (*in vitro*) or with live adults maintained in culture (*ex vivo*). The reaction mixture and cultivation medium, respectively, were analyzed by mass spectrometry and cleavage positions (triangles) in the hormones were identified (see also S3 Table).

We investigated whether SmPOP can interact with peptidic substrates in the environment surrounding the schistosome. NTS or adult schistosomes were incubated in the presence of the fluorogenic peptide substrate Z-Gly-Pro-AMC. Cleavage of the substrate was measured in a microplate reader and was abolished in the presence of the specific POP inhibitor Z-Ala-Pro-CMK (Fig 8A). We also tested whether SmPOP activity is measurable in the excretory/secretory (E/S) products of NTS and adults. For this purpose, E/S products were collected after a two-day cultivation of parasites and SmPOP activity was measured using the same fluorogenic substrate. No significant POP activity was detected in E/S products, demonstrating that SmPOP is not secreted into the cultivation media.

In the next step, we used the above culture assay to measure cleavage by adult parasites of two vasoregulatory proline-containing hormones from the human host, namely angiotensin I and bradykinin. Both hormones were cleaved when added to the cultivation medium and the cleavage occurred specifically after Pro residues as demonstrated by mass spectrometry (Fig 8B). Again, the fragmentation was abolished in the presence of a POP-specific inhibitor Z-Ala-Pro-CMK (but not in the presence of the cysteine peptidase inhibitor E-64; S3 Table). The identified cleavage positions in the hormone sequences were identical with those obtained by *in vitro* fragmentation using rSmPOP.

To conclude, SmPOP, although not secreted from the parasite, can nonetheless interact with physiologically relevant host peptides in the environment.

### SmPOP inhibitors induce deleterious phenotypes in cultured schistosomula

A panel of SmPOP inhibitors was tested at 1 and 10 μM against NTS and the phenotypic responses graded I through III from the most to the least severe (Table 2). The CHO inhibitor, Z-Lys-Pro-CHO, induced a grade I response. Grade II phenotypes were induced by Z-Gly-Pro-CHO, Z-Tyr-Pro-CHO, Z-Arg-Pro-CHO and the CMK inhibitor, Z-Arg-Pro-CMK. The inhibitors Z-Ala-Pro-CHO and Z-Ala-Pro-CMK induced the least severe grade III phenotype. The commercial inhibitors of human POP, Y-29794 and SUAM 14746, induced a grade II response or had no effect, respectively (Table 2).

## Discussion

We identified and functionally characterized a S9-family serine peptidase from the human blood fluke, *S*. *mansoni*. It was denoted SmPOP, *S*. *mansoni* prolyl oligopeptidase, based on its 51% primary sequence identity to human and porcine prolyl oligopeptidases. Also, homology modeling of SmPOP using porcine POP as a structural template revealed that both enzymes share the same spatial architecture and domain structure; specifically, a catalytic peptidase domain with an α/β hydrolase fold and a catalytic triad, and a cylindrical β-propeller domain that covers the active site and defines prolyl oligopeptidase as an oligopeptidase [48].

SmPOP was heterologously expressed in *E*. *coli*, purified as an active peptidase and subjected to a series of biochemical analyses to determine its substrate and inhibitory specificity. Consistent with its classification as a S9-family prolyl oligopeptidase, the enzyme cleaves various oligopeptide substrates in an endopeptidolytic mode at the carboxyl terminus of Pro residues [45]. Cleavage specificity analysis with the positional-scanning substrate library revealed a preference for basic amino acids over hydrophobic or aliphatic amino acids at P2; a Pro residue at P2 was unfavorable. A similar S2 subsite specificity profile was obtained for human POP (Fig 4).

rSmPOP was effectively inhibited by the general serine peptidase inhibitor, diisopropylfluorophosphate [49], but only weakly by inhibitors targeting the S1 family of serine peptidases such as Pefabloc, benzamidine, and BPTI. These data are consistent with the inhibitory specificities of mammalian and trypanosomal POPs [50,51]. The inhibitor specificity of rSmPOP was investigated further using a panel of synthetic prolinal inhibitors that vary at the P2 amino-acid residue (Z-Xaa-Pro-CHO, Table 2). The inhibitor specificity profile mirrored that determined with the positional- scanning substrate library, with the exception of the Pro residue in the P2 position, which generates a good inhibitor but a poor substrate (Z-Pro-Pro-CHO vs. Z-Pro-Pro-ACC, respectively, Table 2 and Fig 4A). Note that Z-Pro-Pro-ACC substrate does not bind effectively in the active site neither as the uncleaved form nor as the hypothetical cleavage product Z-Pro-Pro-OH (as they do not compete with Z-Gly-Pro- substrate). A similar discrepancy was observed for human POP (Table 2 and Fig 4A). Based on the assembled biochemical and structural data, therefore, it is clear that SmPOP and its mammalian orthologs are almost identical in their catalytic specificity profiles suggesting a strong evolutionary conservation of function and structure.

The panel of SmPOP inhibitors was further evaluated for their anti-schistosomal effects against NTS in culture. These tests demonstrated that some of the investigated inhibitors induced deleterious phenotypes or death. Although, interactions other than with the specific target protein cannot be excluded, the data encourage the search for small molecule inhibitors of SmPOP. Inhibitors of human POP are currently being examined as drug leads in several neurological disorders such as depression, Alzheimer’s disease and amnesia, and a number are in preclinical and clinical trials as nootropics (for review see [44]). POP is also of interest for the treatment of celiac sprue, an inflammatory disease of the small intestine caused by ingesting proline-rich gluten [39].

The prolyl oligopeptidases Tc80 and Tb80 from the protozoan parasites *Trypanosoma cruzi* and *T*. *brucei*, respectively, are secreted and can degrade host extracellular-matrix (ECM) proteins such as proline-rich collagens I and IV [23,51]. Tc80’s ability to ability to degrade of ECM components contributes directly to the invasion of mammalian cells by *T*. *cruzi* trypomastigotes [22]. In contrast, SmPOP cannot degrade protein substrates, including collagens, even though it has about 40% identity with trypanosomal POPs (S1 Table). Further, SmPOP is not found in *S*. *mansoni* E/S products suggesting that it is not secreted by schistosomes, a finding consistent with the absence of the signal peptide in the SmPOP sequence. The data would therefore indicate that the trypanosomal POPs possess different physiological functions from those postulated below for the schistosomal enzyme.

By RT-qPCR and substrate analysis, SmPOP is expressed in those developmental stages parasitizing the definitive mammalian host (adults, NTS and eggs). By immunolocalization with a monospecific rabbit antibody SmPOP is distributed in the tegument (males) and parenchyma of NTS and adult schistosomes. The enzyme is absent from the gastrodermis and gut lumen suggesting that the enzyme does not contribute to the digestion of ingested blood proteins. The antibody signal was significantly greater in male worms in accordance with the activity profiling of worm extracts, whereby male worm extracts displayed 5–6 times greater SmPOP specific enzymatic activity than females (S6 Fig). Intriguingly, SmPOP is found in the male tegument, not least in the tubercles, but is apparently absent from the female tegument. This suggests that SmPOP may have male-specific peptidolytic functions at the host–parasite interface and/or at the male-female interface. As noted above, the enzyme seems not to be secreted by the parasite yet, via contact with the endothelium of the host vasculature, may exert localized effects on vascular physiology, including the degradation of vasoactive peptides (see below). A similar localization in the tegument and parenchyma was previously noted for the *S*. *mansoni* cysteine peptidase cathepsin B2 for which physiological function(s) are not yet known [52].

We demonstrate that the schistosome parasite can cleave the vasoregulatory peptides, angiotensin I and bradykinin, when co-incubated *in vitro* and that the activity is due to SmPOP as indicated by mass spectrometry and specific inhibition by a POP inhibitor. Angiotensin I is produced by the renin-angiotensin system which is the primary physiological regulator of blood pressure, sodium balance and fluid volume [53]. SmPOP converts angiotensin I (precursor of the main vasoconstrictor angiotensin II) to the vasodilatory angiotensin-(1–7). Angiotensin-(1–7) also inhibits cell proliferation, angiogenesis, fibrosis, and inflammation [54,55]. Bradykinin is generated by the kallikrein-kinin system which also participates in the regulation of blood pressure [53]. Bradykinin is a potent vasodilator, promotes natriuresis, diuresis and inflammation. Proteolytic cleavage by SmPOP inactivates this hormone. The possible contribution, therefore, by a tegument-localized SmPOP to the modulation or dysregulation of both these, and possibly, other, homeostatic systems is conceivable whereby cleavage of the pro-inflammatory and vasoconstrictory angiotensin I and pro-inflammatory bradykinin may provide a survival benefit to the schistosome during its residence in and movement through the venous blood system. Follow-up *in vivo* studies will examine these possibilities in more detail.

## Supporting Information

S1 Fig Examples of phenotypes induced in cultured NTS by POP inhibitors listed in Table 2. NTS were incubated up to four days in Basch Medium 169 in the presence of inhibitors (for details see Methods). Images were captured using a Zeiss Axiovert 40 C inverted microscope (10x objective) and a Zeiss AxioCam MRc digital camera controlled by AxioVision 40 (version 4.8.1.0) software. Scale bar = 150 μm.
(TIF)

S2 Fig A multiple sequence alignment of *S*. *mansoni* prolyl oligopeptidase (SmPOP) with selected POPs from other blood-feeding parasites and mammalian POPs. Parasite POPs: SmPOP (S. mansoni, GenBank accession number KF956809), *Pediculus humanus* (P. humanus, XP_002430998), *Aedes aegypti* (A. aegypti, Q16WP2), *Ixodes scapularis* (I. scapularis, B7PDF5), *Toxoplasma gondi* (T. gondi, XP_002369249), *Trypanosoma cruzi* (T. cruzi, AAQ04681) and *Leishmania infantum* (L. infantum, CAM72491.1). Mammalian POPs: human (H. sapiens, P48147) and porcine (S. scrofa, P23687). Catalytic-triad residues (Ser, Asp and His) are indicated in red; those residues identical with those of SmPOP are shaded in gray. The residue numbering corresponds to the SmPOP sequence and its color coding refers to the domain structure of POPs consisting of the N-terminal segment (green), the β-propeller domain (cyan) and the peptidase catalytic domain (magenta).
(TIF)

S3 Fig The maximum-likelihood phylogenetic tree displaying the evolutionary relationship of SmPOP to selected POPs from other organisms. A multiple alignment of SmPOP with 35 other POP protein sequences was performed using Clustal X 2.0 and the default parameters. The resulting alignment was edited to exclude ambiguous regions by the BioEdit 7.0 editing software. The phylogenetic analysis of the multiple alignment was performed using the maximum likelihood method in PAUP 4.0. The tree was visualized using the Treeview 1.6.6. program. Bootstrap values with 100 repeats are shown at the nodes. GenBank or HelmDB accession numbers of the aligned sequences are indicated. SmPOP is underlined in red and bold type faces. **GenBank accession numbers: Kinetoplastida—**CAM72491 *Leishmania infantum*, *AAQ04681 Trypanosoma cruzi*, CAD42967 *Trypanosoma brucei; Apicomplexa—*XP_002369249 *Toxoplasma gondii*; **Bacteria—**WP_007903716 *Ktedonobacter racemifer*, WP_008080757 *Vibrio sinaloensis*, WP_012088900 *Shewanella baltica*, WP_013626821 *Planctomyces brasiliensis*, WP_011140683 *Gloeobacter violaceus*, WP_011612632 *Trichodesmium erythraeum*, EHJ13806 *Crocosphaera watsonii*, WP_012411436 *Nostoc punctiforme*, WP_006634458 *Microcoleus vaginatus*; **Arthropoda—**XP_002430998 *Pediculus humanus*, B7PDF5 *Ixodes scapularis*, Q16WP2 *Aedes aegypti*, B0W4N7 *Culex quinquefasciatus*, EFN76622 *Harpegnathos saltator*, EFN66352 *Camponotus floridanus;* **Plantae—**A9SA32 *Physcomitrella patens*, XP_002285910 *Vitis vinifera*, ACG43067 *Zea mays*; **Vertebrata—**C0HBI8 *Salmo salar*, Q503E2 *Danio rerio*, Q6P4W3 *Xenopus tropicalis*, F1NUS2 *Gallus gallus*, O70196 *Rattus norvegicus*, Q9QUR6 *Mus musculus*, F1PHX2 *Canis familiaris*, P48147 *Homo sapiens*, Q9XTA2 *Bos taurus*, P23687 *Sus scrofa;* **Trematoda—**KF956809 Schistosoma mansoni. **HelmDB accession numbers: Nematoda—**Asuu161668 *Ascaris suum;* **Trematoda—**Fhep110926 *Fasciola hepatica*, Shae172866 *Schistosoma haematobium*.
(TIF)

S4 Fig Recombinant SmPOP does not digest host-derived macromolecular protein substrates. Human serum albumin (HSA), human collagens type I and IV (Col I and Col IV) and human hemoglobin (Hb) were incubated for 12 h in the presence or absence of rSmPOP. The reaction mixtures were subjected to SDS-PAGE (HSA, Col I and Col IV) or Tricine-SDS-PAGE (Hb) and protein stained. For details, see Methods.
(TIF)

S5 Fig The localization of SmPOP in *S*. *mansoni* newly transformed schistosomula (NTS). Parasites were fixed and probed with anti-SmPOP IgG (A) or a pre-immune IgG from the same rabbit (B). Anti-rabbit IgG Alexa 594-was used as the secondary antibody (red). DAPI was used to label the nuclear DNA (blue) and the fluorescent signals were merged with differential interference contrast (DIC). The greatest red fluorescence is localized to the surface (tegument) with a low diffuse signal in the parenchyma (SmPOP in A). The gut is negative for the SmPOP signal (the asterisk). NTS probed with pre-immune IgG lack any visible fluorescence in the red channel (SmPOP in B). Scale bar = 50 μm.
(TIF)

S6 Fig The SmPOP activity in *S*. *mansoni* adult males and females. SmPOP activities were measured in protein extracts of adult males and females (green bars) or in cultivation medium post incubation with live parasites (red bars). Z-Gly-Pro-AMC was used as the fluorogenic substrate. SmPOP activity (which was sensitive to inhibition by the specific POP inhibitor, Z-Pro-Pro-CHO) was normalized to protein content of extracts or number of worms used.
(TIF)

S1 Table Identity matrix of POP amino acid sequences aligned in S1 Fig. (PDF)

S2 Table Identification of native SmPOP by mass spectrometry. (PDF)

S3 Table The fragmentation of peptide hormones by live *S*. *mansoni* adults. (PDF)
