# Supplementary figures and images for: Prolyl Oligopeptidase from the Blood Fluke Schistosoma mansoni: From Functional Analysis to Anti-schistosomal Inhibitors
Source: PLoS Negl Trop Dis. 2015 Jun 3;9(6):e0003827. doi: 10.1371/journal.pntd.0003827 (PMC4454677; doi:10.1371/journal.pntd.0003827)

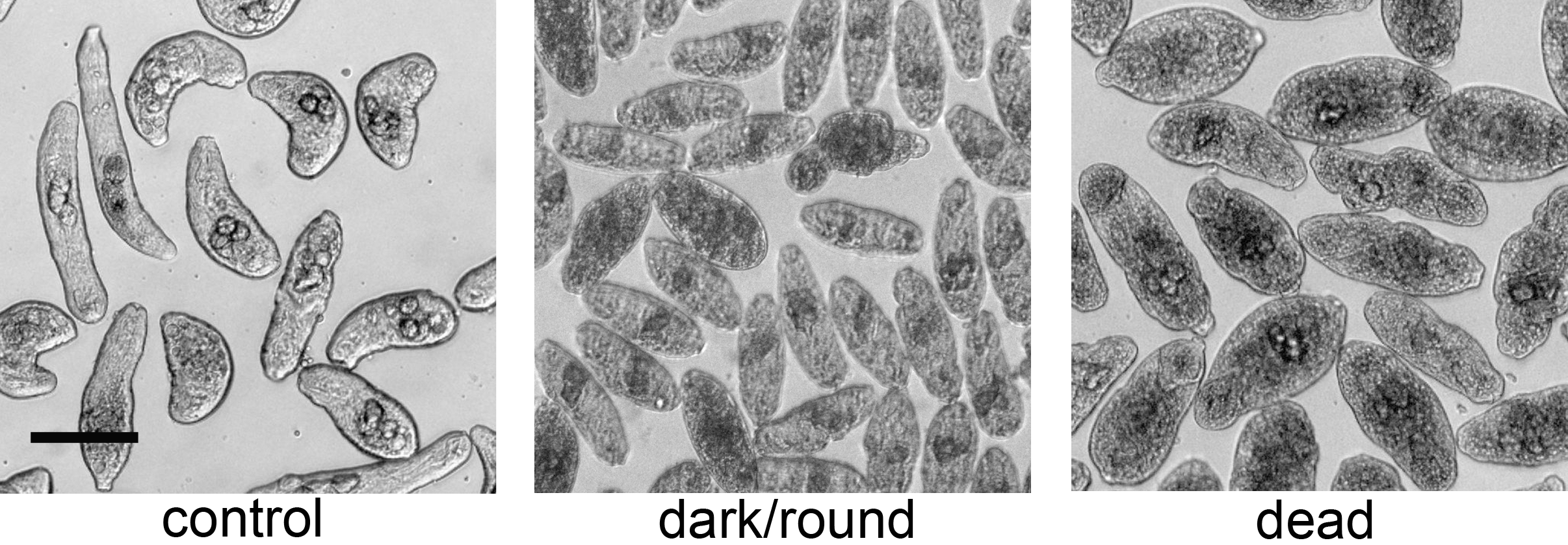

Supplement: S1 Fig — NTS were incubated up to four days in Basch Medium 169 in the presence of inhibitors (for details see Methods). Images were captured using a Zeiss Axiovert 40 C inverted microscope (10x objective) and a Zeiss AxioCam MRc digital camera controlled by AxioVision 40 (version 4.8.1.0) software. Scale bar = 150 μm. (TIF) [file pntd.0003827.s001.tif]

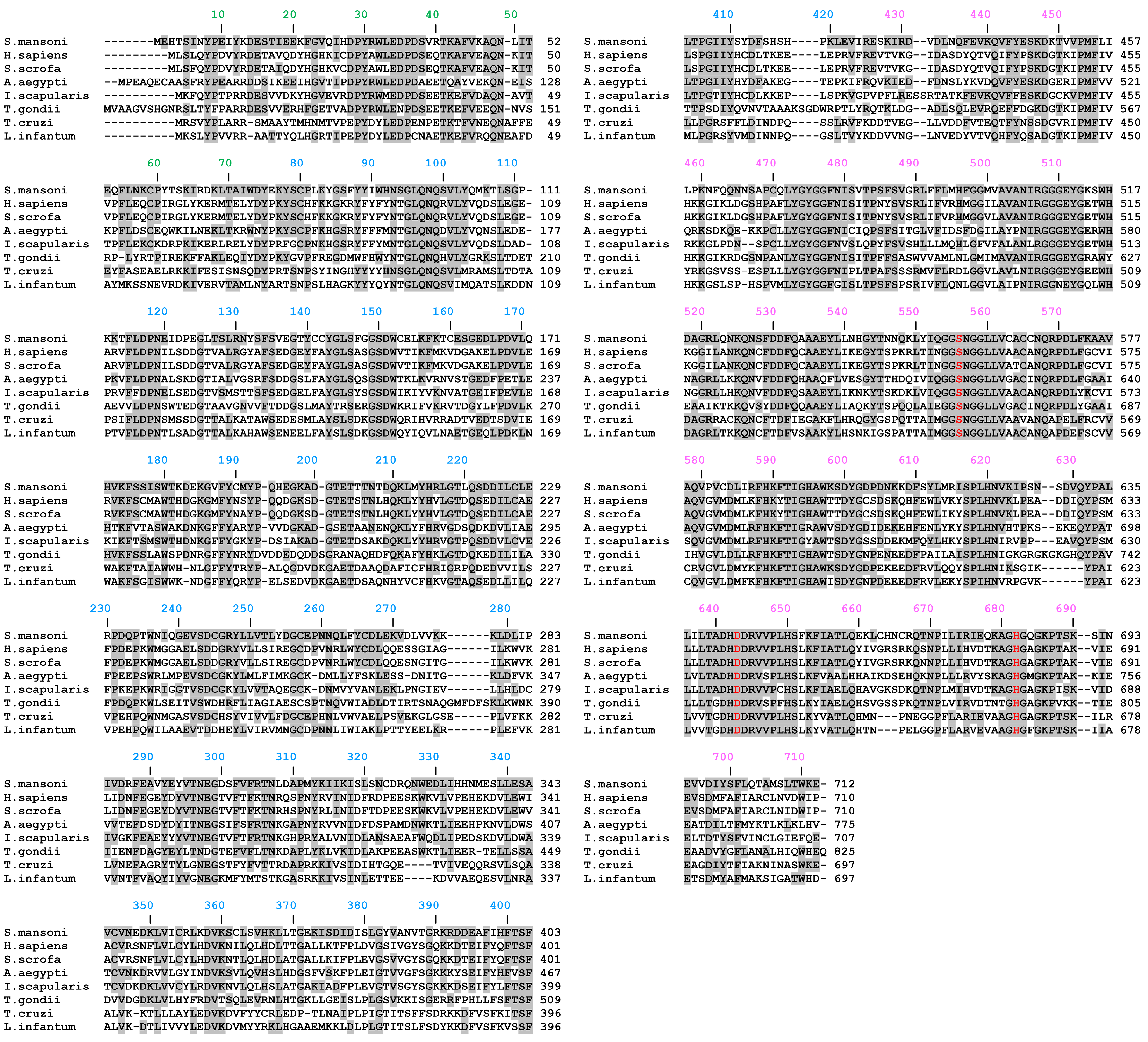

Supplement: S2 Fig — Parasite POPs: SmPOP (S. mansoni, GenBank accession number KF956809), Pediculus humanus (P. humanus, XP_002430998), Aedes aegypti (A. aegypti, Q16WP2), Ixodes scapularis (I. scapularis, B7PDF5), Toxoplasma gondi (T. gondi, XP_002369249), Trypanosoma cruzi (T. cruzi, AAQ04681) and Leishmania infantum (L. infantum, CAM72491.1). Mammalian POPs: human (H. sapiens, P48147) and porcine (S. scrofa, P23687). Catalytic-triad residues (Ser, Asp and His) are indicated in red; those residues identical with those of SmPOP are shaded in gray. The residue numbering corresponds to the SmPOP sequence and its color coding refers to the domain structure of POPs consisting of the N-terminal segment (green), the β-propeller domain (cyan) and the peptidase catalytic domain (magenta). (TIF) [file pntd.0003827.s002.tif]

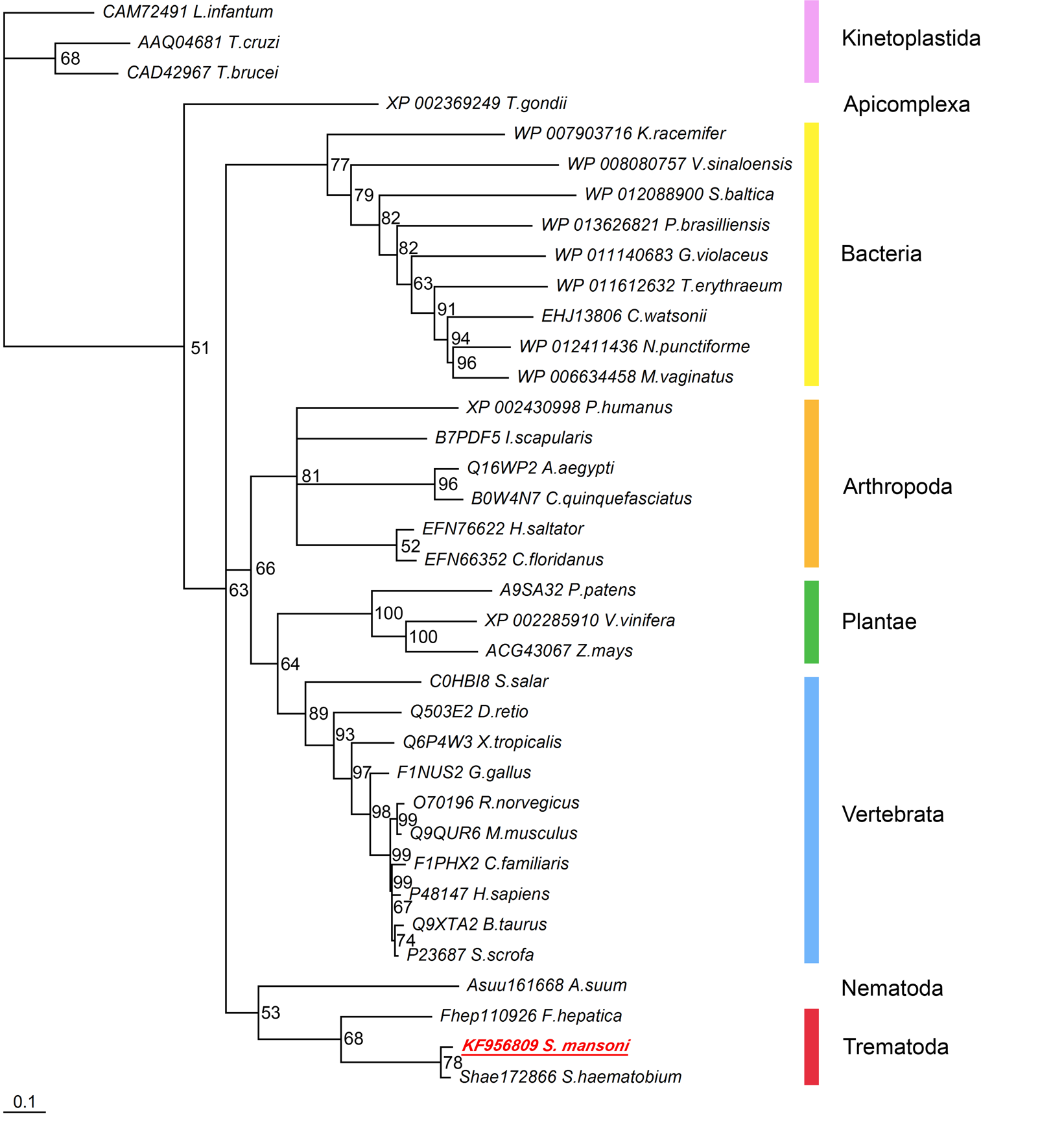

Supplement: S3 Fig — A multiple alignment of SmPOP with 35 other POP protein sequences was performed using Clustal X 2.0 and the default parameters. The resulting alignment was edited to exclude ambiguous regions by the BioEdit 7.0 editing software. The phylogenetic analysis of the multiple alignment was performed using the maximum likelihood method in PAUP 4.0. The tree was visualized using the Treeview 1.6.6. program. Bootstrap values with 100 repeats are shown at the nodes. GenBank or HelmDB accession numbers of the aligned sequences are indicated. SmPOP is underlined in red and bold type faces. GenBank accession numbers: Kinetoplastida—CAM72491 Leishmania infantum, AAQ04681 Trypanosoma cruzi, CAD42967 Trypanosoma brucei; Apicomplexa—XP_002369249 Toxoplasma gondii; Bacteria—WP_007903716 Ktedonobacter racemifer, WP_008080757 Vibrio sinaloensis, WP_012088900 Shewanella baltica, WP_013626821 Planctomyces brasiliensis, WP_011140683 Gloeobacter violaceus, WP_011612632 Trichodesmium erythraeum, EHJ13806 Crocosphaera watsonii, WP_012411436 Nostoc punctiforme, WP_006634458 Microcoleus vaginatus; Arthropoda—XP_002430998 Pediculus humanus, B7PDF5 Ixodes scapularis, Q16WP2 Aedes aegypti, B0W4N7 Culex quinquefasciatus, EFN76622 Harpegnathos saltator, EFN66352 Camponotus floridanus; Plantae—A9SA32 Physcomitrella patens, XP_002285910 Vitis vinifera, ACG43067 Zea mays; Vertebrata—C0HBI8 Salmo salar, Q503E2 Danio rerio, Q6P4W3 Xenopus tropicalis, F1NUS2 Gallus gallus, O70196 Rattus norvegicus, Q9QUR6 Mus musculus, F1PHX2 Canis familiaris, P48147 Homo sapiens, Q9XTA2 Bos taurus, P23687 Sus scrofa; Trematoda—KF956809 Schistosoma mansoni. HelmDB accession numbers: Nematoda—Asuu161668 Ascaris suum; Trematoda—Fhep110926 Fasciola hepatica, Shae172866 Schistosoma haematobium. (TIF) [file pntd.0003827.s003.tif]

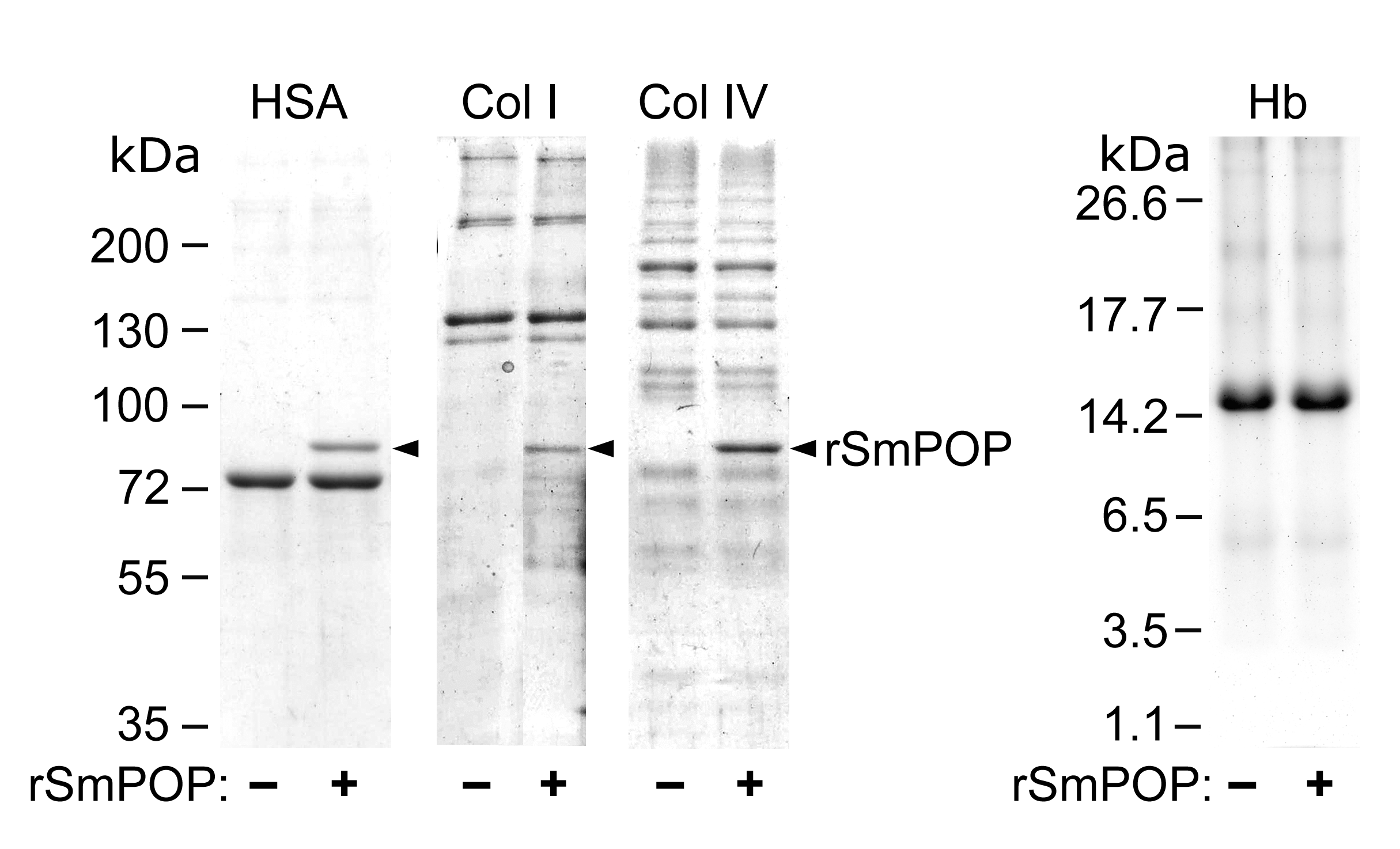

Supplement: S4 Fig — Human serum albumin (HSA), human collagens type I and IV (Col I and Col IV) and human hemoglobin (Hb) were incubated for 12 h in the presence or absence of rSmPOP. The reaction mixtures were subjected to SDS-PAGE (HSA, Col I and Col IV) or Tricine-SDS-PAGE (Hb) and protein stained. For details, see Methods. (TIF) [file pntd.0003827.s004.tif]

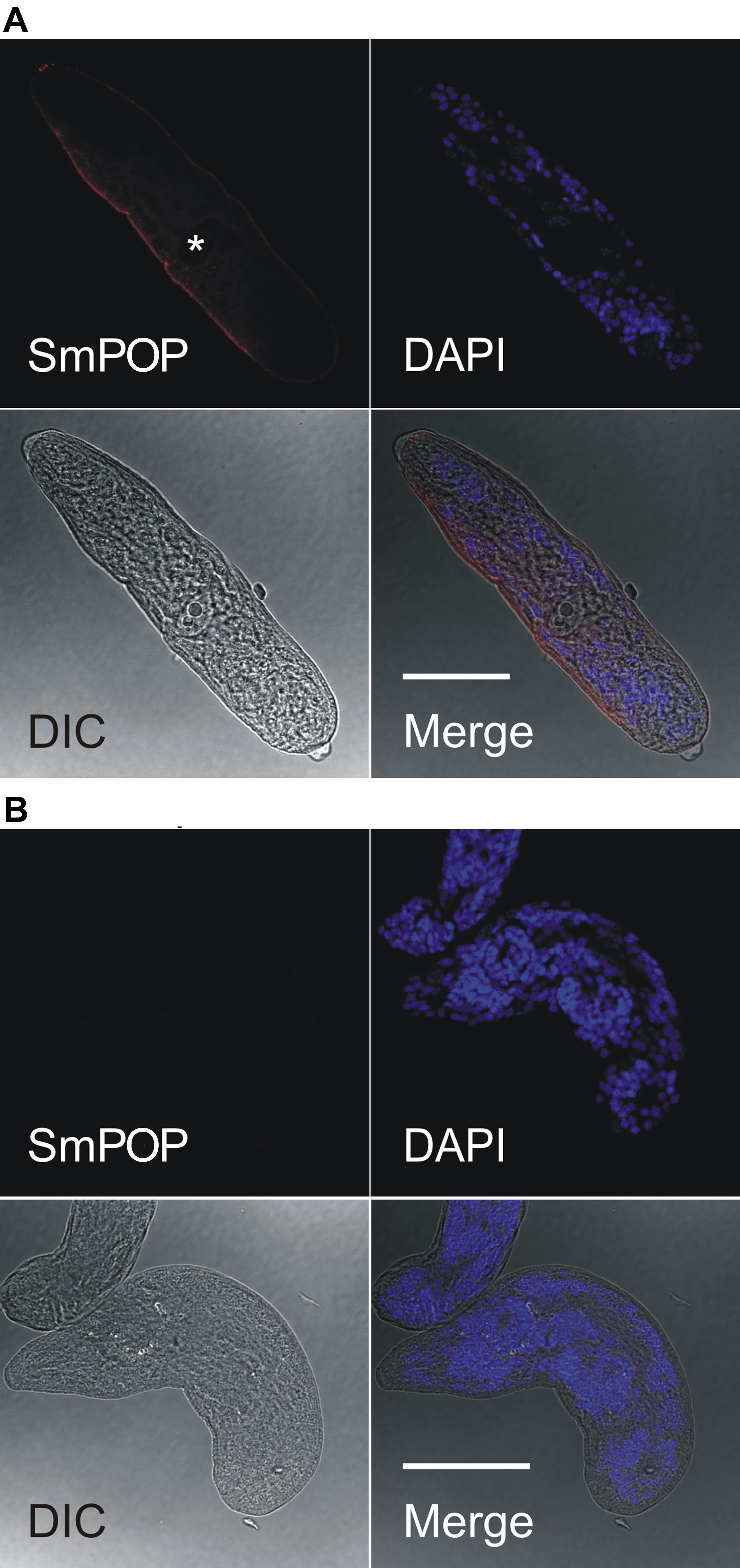

Supplement: S5 Fig — Parasites were fixed and probed with anti-SmPOP IgG (A) or a pre-immune IgG from the same rabbit (B). Anti-rabbit IgG Alexa 594-was used as the secondary antibody (red). DAPI was used to label the nuclear DNA (blue) and the fluorescent signals were merged with differential interference contrast (DIC). The greatest red fluorescence is localized to the surface (tegument) with a low diffuse signal in the parenchyma (SmPOP in A). The gut is negative for the SmPOP signal (the asterisk). NTS probed with pre-immune IgG lack any visible fluorescence in the red channel (SmPOP in B). Scale bar = 50 μm. (TIF) [file pntd.0003827.s005.tif]

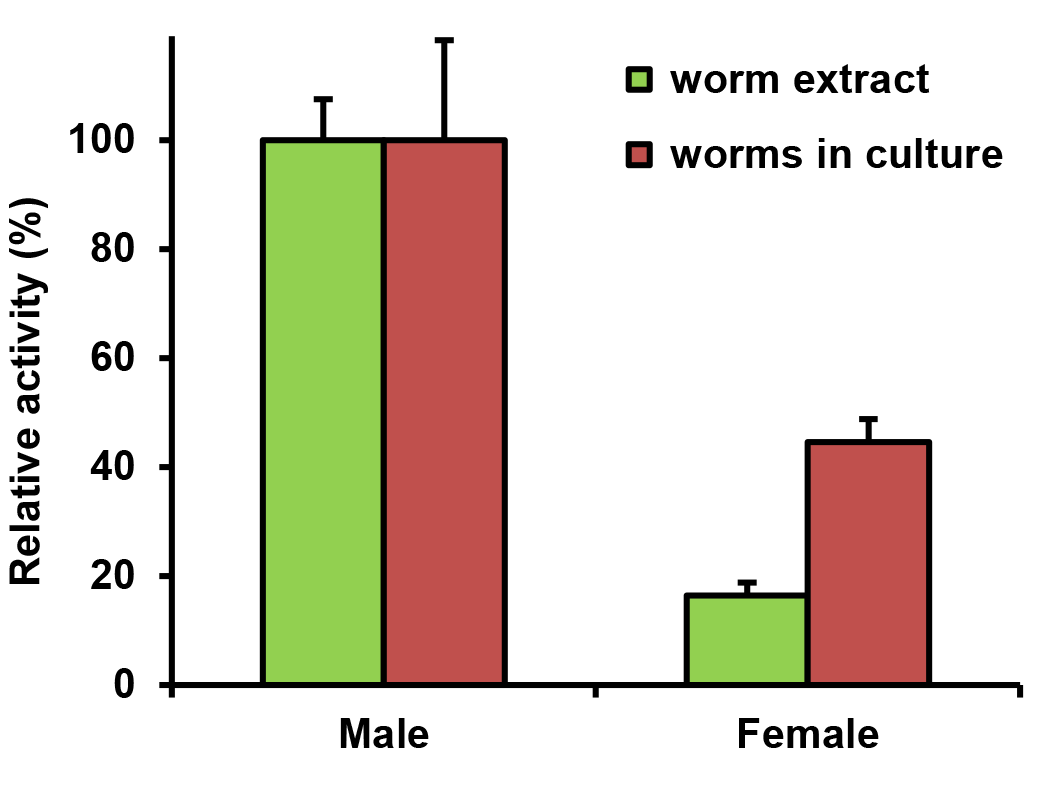

Supplement: S6 Fig — SmPOP activities were measured in protein extracts of adult males and females (green bars) or in cultivation medium post incubation with live parasites (red bars). Z-Gly-Pro-AMC was used as the fluorogenic substrate. SmPOP activity (which was sensitive to inhibition by the specific POP inhibitor, Z-Pro-Pro-CHO) was normalized to protein content of extracts or number of worms used. (TIF) [file pntd.0003827.s006.tif]
