# Supplementary material for: Prolyl Oligopeptidase from the Blood Fluke Schistosoma mansoni: From Functional Analysis to Anti-schistosomal Inhibitors
Source: PLoS Negl Trop Dis. 2015 Jun 3;9(6):e0003827. doi: 10.1371/journal.pntd.0003827 (PMC4454677; doi:10.1371/journal.pntd.0003827)
Supplement: S1 Table — (PDF) [file pntd.0003827.s007.pdf]

## S1 Table. Identity matrix of POP amino acid sequences aligned in S1 Figure.

| Sequence[a] | HsPOP | SsPOP | PhPOP | AaPOP | IsPOP | TgPOP | TcPOP | LiPOP |
|---|---|---|---|---|---|---|---|---|
| **SmPOP** | 51 | 51 | 49 | 50 | 51 | 45 | 41 | 41 |
| **HsPOP** | -- | 97 | 55 | 55 | 59 | 49 | 43 | 44 |
| **SsPOP** | | -- | 54 | 55 | 60 | 49 | 43 | 44 |
| **PhPOP** | | | -- | 53 | 53 | 45 | 41 | 40 |
| **AaPOP** | | | | -- | 56 | 43 | 43 | 44 |
| **IsPOP** | | | | | -- | 49 | 43 | 44 |
| **TgPOP** | | | | | | -- | 41 | 41 |
| **TcPOP** | | | | | | | -- | 64 |

[a] POP sequences from *S. mansoni* (SmPOP), *H. sapiens* (HsPOP), *S. scrofa* (SsPOP), *P. humanus* (PhPOP), *A. aegypti* (AaPOP), *I. scapularis* (IsPOP), *T. gondi* (TgPOP), *T. cruzi* (TcPOP) and *L. infantum* (LiPOP).
