# Supplementary material for: Prolyl Oligopeptidase from the Blood Fluke Schistosoma mansoni: From Functional Analysis to Anti-schistosomal Inhibitors
Source: PLoS Negl Trop Dis. 2015 Jun 3;9(6):e0003827. doi: 10.1371/journal.pntd.0003827 (PMC4454677; doi:10.1371/journal.pntd.0003827)
Supplement: S2 Table — (PDF) [file pntd.0003827.s008.pdf]

S2 Table. Identification of native SmPOP by mass spectrometry.

Peptide sequence	Peptide position (AA residues)	Theoretical peptide mass $[M+H]^+$ (Da)	Measured peptide mass $[M+H]^+$ (Da)	Delta (ppm)
FGVQIHDPYR	22-31	1230.6145	1230.6135	-0.79
VKAQNLITEQF	45-55	1289.6980	1289.6913	-5.21
FLDPNEIDPEGLTSLR	115-130	1814.9050	1814.9034	-0.87
FSSISWTK	175-182	954.4811	954.4787	-2.49
TSFLTPGIIY	401-410	1110.5961	1110.5958	-0.22
DVDLNQFEVK	431-440	1205.5928	1205.5885	-3.54
HNVKIPSNSDVQYPALL	620-636	1893.9949	1894.0009	3.16

The protein extract of *S. mansoni* adults was subjected to LC-MS/MS analysis to identify peptide fragments specific for SmPOP. The protein extract was precipitated with acetone and digested with trypsin or chymotrypsin. Digests were analyzed on a UltiMate 3000 RSLCnano system (Dionex) coupled to a TripleTOF 5600 mass spectrometer with a NanoSpray III source (AB Sciex). The first dimension column was an Acclaim PepMap 100 column (5 µm, 2 cm × 100 µm ID, Dionex) and the second dimension column was an Acclaim PepMap 100 analytical column (3 µm, 15 cm × 75 µm ID, Dionex). The mass data were processed by the ProteinPilot Software 4.5 (AB Sciex).
