# Supplementary material for: Prolyl Oligopeptidase from the Blood Fluke Schistosoma mansoni: From Functional Analysis to Anti-schistosomal Inhibitors
Source: PLoS Negl Trop Dis. 2015 Jun 3;9(6):e0003827. doi: 10.1371/journal.pntd.0003827 (PMC4454677; doi:10.1371/journal.pntd.0003827)
Supplement: S3 Table — (PDF) [file pntd.0003827.s009.pdf]

S3 Table. The fragmentation of peptide hormones by live *S. mansoni* adults.

Inhibitor Treatment	Hormone	Identified peptide	Theoretical peptide mass $[M+H]^+$ (Da)	Measured peptide mass $[M+H]^+$ (Da)	Fragment (aa residues)
**No Inhibitor**	**Angiotensin I**	D R V Y I H P F H L	1296.6853	1296.7126	1-10 (uncleaved)
		D R V Y I H P	899.4739	899.4784	1-7
	**Bradykinin**	R P P G F S P F R	1060.5692	1060.5922	1-9 (uncleaved)
		R P P G F S P	757.3997	757.3717	1-7
		G F S P F R	710.3626	710.3131	4-9
**E-64**	**Angiotensin I**	D R V Y I H P F H L	1296.6853	1296.7163	1-10 (uncleaved)
		D R V Y I H P	899.4739	899.4788	1-7
	**Bradykinin**	R P P G F S P F R	1060.5692	1060.5894	1-9 (uncleaved)
		R P P G F S P	757.3997	757.3730	1-7
		G F S P F R	710.3626	710.3341	4-9
**Z-Ala-Pro-CMK**	**Angiotensin I**	D R V Y I H P F H L	1296.6853	1296.7068	1-10 (uncleaved)
	**Bradykinin**	R P P G F S P F R	1060.5692	1060.5894	1-9 (uncleaved)

The peptide hormones were incubated with live adult schistosomes in culture in the presence of given inhibitor and the cultivation medium was analyzed after 16 h by MALDI-TOF mass spectrometry to identify any resulting hydrolytic fragments. For technical details, see the Materials and Methods and Figure 8.
